# Cost‐Effectiveness of Personalised Nutrition in Adults With Overweight and Obesity: PREVENTOMICS Studies in Poland and the UK

**DOI:** 10.1111/jhn.70071

**Published:** 2025-06-02

**Authors:** Milanne Maria Johanna Galekop, Philip C. Calder, Ella J. Baker, Joanna Góralska, Urszula Raźny, Malgorzata Malczewska‐Malec, Carin A. Uyl‐de Groot, William Ken Redekop

**Affiliations:** ^1^ Erasmus School of Health Policy and Management, Erasmus University Rotterdam Rotterdam The Netherlands; ^2^ School of Human Development and Health, Faculty of Medicine, University of Southampton Southampton UK; ^3^ NIHR Southampton Biomedical Research Centre, University Hospital Southampton NHS Foundation Trust and University of Southampton Southampton UK; ^4^ Department of Clinical Biochemistry, Genetics and Nutrigenomics Chair in Clinical Biochemistry, Jagiellonian University Medical College Krakow Poland

**Keywords:** cost‐effectiveness analysis, obesity, overweight, personalised nutrition

## Abstract

**Background:**

We assessed the cost‐effectiveness of personalised nutrition in adults with overweight/obesity in Poland and the United Kingdom (UK) using the results of two randomised controlled trials (RCTs).

**Methods:**

The 4‐month RCTs compared three interventions: personalised plan (PP) plus behavioural change (PP+B), PP only, and a control. Outcomes included body mass index (BMI), health‐related quality of life (EQ‐5D‐5L), quality‐adjusted life years (QALYs), and costs (2020 British pounds). A Markov model estimated lifetime cost‐effectiveness. Different sensitivity analyses were performed.

**Results:**

Participants were randomised to PP+B, PP, and control groups in Poland (*n* = 89, *n* = 88, *n* = 88) and the UK (*n* = 20, *n* = 19, *n* = 15). Comparing BMI reductions of PP+B and PP with control in both countries showed no significant differences, but wide confidence intervals (CIs) were observed (e.g., PP+B vs. control—Poland: −0.20, 95% CI: −0.86, 0.45 kg/m^2^; UK: −0.80, 95% CI: −1.60, 0.00 kg/m^2^). Lifetime analysis suggested potential cost‐effectiveness for PP+B in Poland (£20,404 per QALY gain), and for PP+B (£13,006 per QALY) and PP (£12,222 per QALY) in the UK, since these figures were lower than the willingness‐to‐pay thresholds (£34,000 in Poland and £20,000 in the UK). PP in Poland was dominated by control, but sensitivity analyses suggested potential cost‐effectiveness.

**Conclusions:**

The PREVENTOMICS interventions may offer a cost‐effective approach to reduce weight and avoid its related complications in both countries. Future studies should be larger and/or longer to reduce uncertainty about effectiveness.

**Clinical Trial Registration Numbers:** Poland ISRCTN51509551 and the UK ISRCTN46063864.

## Summary

- This study examined the cost‐effectiveness of advanced personalised nutrition interventions developed during the PREVENTOMICS project in Poland and the UK.
- Results showed that personalised nutrition may be a cost‐effective approach for weight reduction and better health outcomes in adults with overweight/obesity.
- Larger, long‐term trials are needed to validate findings and assess sustained behavioural changes.

## 1 Introduction

Obesity is a major public health problem that carries a significant health and financial impact [1, 2]. In 2016, there were globally more than 1.9 billion adults with overweight or obesity [1]. The United Kingdom (UK) has one of the highest obesity rates, where approximately 28% adults live with obesity and 36% are considered overweight (but not obese) [2]. In Poland, over half of the adult population has excess body weight (58%); 23% were living with obesity and 35% with overweight [2]. Overweight/obesity is estimated to reduce mean life expectancy by approximately 2.7 years in the UK and 3.9 years in Poland [2]. This shorter life expectancy is partly due to the direct effects of overweight and obesity, but it is mainly caused by associated conditions like heart disease, stroke, and Type 2 diabetes. Additionally, overweight and obesity significantly contribute to healthcare costs [2]. In the UK and Poland, overweight accounted for 8.4% and 6.4% of the total healthcare expenditure, respectively [2]. This impact extends to productivity losses, with approximately 944,000 full‐time workers absent annually in the UK due to overweight/obesity‐related productivity issues [2]. These productivity losses are an example of indirect costs, which numerous studies have demonstrated to be higher than the direct costs of obesity [3, 4, 5]. For example, Dee et al. [4] showed in their systematic review that indirect costs comprise between 54% and 59% of the total costs of overweight and obesity.

The health and economic impact of overweight and obesity highlights the need to prevent and manage obesity and its related health conditions. Nutrition is a widely recognised element in the prevention and treatment of obesity, as it is a modifiable risk factor [6, 7]. However, to enhance its effectiveness, guidelines and policies need to shift from a population‐based approach to a more individual approach (i.e., personalised nutrition), given the individual variations in nutritional response [7, 8, 9]. Personalised nutrition uses information on individual characteristics, such as genotype, phenotype, behaviour, and preferences, to create targeted nutritional advice, products, or services [9]. Several studies in this field have revealed associations between genetic factors and food metabolism, nutrition needs, dietary preferences, and disease outcomes [9, 10, 11].

Because of the potential of personalised nutrition to improve health and decrease the economic impact of diet‐related diseases, efforts are being made to develop personalised interventions that can achieve that potential [12]. The PREVENTOMICS project (EU Horizon 2020: No. 818318) aimed to develop and implement a system that enabled personalised nutrition advice using omics sciences to reduce the risk of diet‐related diseases [13, 14]. The project developed a platform with a decision support system (DSS) that integrates multiple variables, including data on participants’ genetics, metabolic profile, behaviour, and body composition, to generate personalised recommendations using machine learning techniques [13]. This platform was integrated into different digital applications, including the MetaDieta app for nutrition professionals in Poland and the UK, which were assessed in clinical trials conducted in both countries [15, 16]. In these trials, participants with overweight or obesity were randomly assigned to three groups: (1) personalised plan (PP) + behavioural change (PP+B) group: dieticians used the MetaDieta app to generate personalised diets and provided behavioural change prompts; (2) PP group: similar to PP+B, but without behavioural change prompts; (3) Control group: standard dietetic advice from dieticians.

Since effectiveness and cost‐effectiveness are crucial factors in healthcare decision‐making, it is vital to evaluate both within the context of the PREVENTOMICS interventions (i.e., PP+B and PP) [17]. Assessing the cost‐effectiveness of the personalisation of nutrition is especially important considering the potential of personalised nutrition to enhance health, alongside the potentially high costs related to omics analyses integral to PREVENTOMICS interventions [18, 19]. The estimation of their potential cost‐effectiveness using data from the PREVENTOMICS trials at this early stage (i.e., early cost‐effectiveness analysis) can assist developers and policymakers when making decisions regarding future development, implementation and reimbursement (e.g., ‘stop or go’ decision) [20, 21, 22]. This study, therefore, aims to address the potential cost‐effectiveness of the personalisation of nutrition by comparing PP+B and PP to a control in adults with overweight and obesity in Poland and the UK.

## 2 Methods

### 2.1 Overall Study Design

Data from two single‐blind, randomised, placebo‐controlled trials were used to analyse and calculate long‐term health outcomes, costs, and cost‐effectiveness of personalised nutrition interventions [15, 16]. A health economic analysis plan was developed as part of the PREVENTOMICS proposal; the extended version of this plan is found below. The Consolidated Health Economic Evaluation Reporting Standards (CHEERS) statement was followed [23].

### 2.2 Trial Description

The clinical trials were part of the PREVENTOMICS project and had similar study protocols, lasting 4 months and were conducted in Poland (registration number: ISRCTN51509551) and the UK (registration number: ISRCTN46063864). However, some differences in protocols were observed (e.g., dieticians in the UK stressed the importance of protein intake [in all groups], unlike dieticians in Poland). Participants aged 18–65 years with abdominal obesity (i.e., body mass index [BMI] between 25 and 40 kg/m^2^ and waist circumference > 94 cm for men or > 80 cm for women) [24] were randomly assigned to one of the three intervention arms: PP+B, PP, or control [15, 16]. To accurately measure the isolated effect of personalisation, the control intervention was designed to closely resemble the personalised arms. The control group received general dietary recommendations by the dietician. These were based on European guidelines for obesity management in adults [25]. In contrast, the PP+B and PP groups received personalised dietary advice based on metabolomic, inflammatory, and genetic profiles by the dietician [13]. Participants in the PP+B and PP groups were assigned to metabolic clusters, as indicated by the DSS. These clusters represented disorders in carbohydrate metabolism, lipid metabolism, inflammation, oxidative stress, or gut microbiota. Each participant's dominant cluster served as the basis for generating personalised dietary advice, considering individuals’ dietary habits, behaviour, body composition and physical activity. The MetaDieta software then processed this advice generated by the DSS, enabling dieticians to provide individualised dietary plans to each participant [26, 27]. Participants themselves also used a MetaDieta app to support dietary compliance, to monitor food intake and to contact the dietician, with whom, additionally, one consultation per month was scheduled. The PP+B group also received behavioural change prompts through the MetaDieta app [13, 26, 28]. More details about the trials are available on the trial registration websites [15, 16].

### 2.3 Model Structure

**Figure 1:**
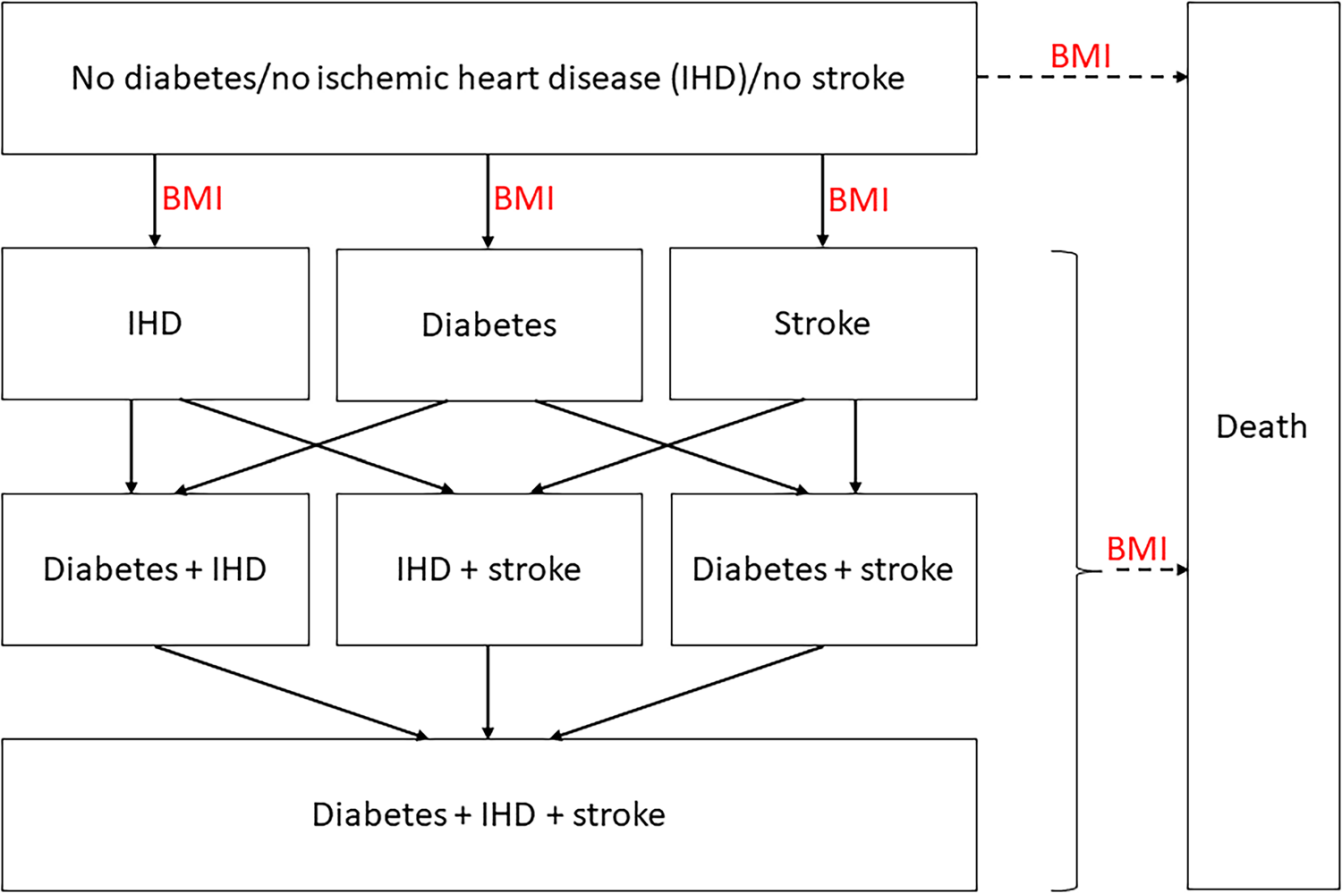
Structure of the Markov model for obesity as described by Hoogendoorn et al. [5] BMI, body mass index; IHD, ischemic heart disease.

A validated obesity economic model was used to extrapolate the 4‐month trial results over a lifetime horizon, since the trial period was considered too short to estimate all relevant costs and effects. The model, implemented as a Markov model, incorporated the occurrence of obesity‐related diseases and had a cycle length of 1 year [5]. Figure 1 presents the model's structure, where each rectangle represents a distinct health state. The initial health state was ‘no diabetes/ischemic heart disease (IHD)/stroke’ for a cohort with obesity, characterised by mean age, mean BMI, and male/female ratio based on the trial populations.

The cohort's progression was simulated over time by considering the development of the different diseases and death. This simulation was conducted once in a base‐case scenario and further expanded through scenario analyses and probabilistic sensitivity analyses (PSAs), as detailed in subsection 2.6 ‘Sensitivity analyses’. The incidence of diseases and mortality rates were contingent upon various factors, including age, sex, BMI, and the prevailing health state [5]. Mortality in the diabetes state included both diabetes‐specific mortality and mortality from other causes. Given the high mortality risk associated with stroke and IHD, including myocardial infarction (MI), at the time of their occurrence, mortality was further categorised into case fatality, mortality specifically attributable to IHD or stroke, and mortality from other causes.

In the model, BMI was treated as a continuous variable [5]. All analyses were conducted using R and RStudio (version Ri386 3.6.1/Rx64 3.6.1). Comprehensive details regarding the model can be found elsewhere [5].

### 2.4 Model Inputs

Model inputs for extrapolating the trial effects, such as demographic and epidemiological parameters (as input for transition probabilities), effectiveness parameters, and cost parameters, were obtained from various sources (see Table 1) [5].

**Table 1:** Model inputs specified by country^a^.

	Poland			UK			Both countries
Parameter	Deterministic value	Sensitivity analysis range (CI or assumption)	Source	Deterministic value	Sensitivity analysis range (CI or assumption)	Source	Distribution
**General**							
Time horizon, years	Lifetime	—	—	Lifetime	—	—	—
Cycle length	1 year	—	—	1 year	—	—	—
RRs for association BMI and diabetes, IHD and stroke	RR varied by BMI, specified by age	—	GBD [29]	RR varied by BMI, specified by age	—	GBD [29]	Normal distribution
RRs for the co‐occurrence of diseases	RR specified by age and sex	—	Different sources [30, 31, 32]	RR specified by age and sex	—	Different sources [30, 31, 32]	Fixed
RRs for association BMI and all‐cause mortality	RR varied by BMI, specified by sex	—	Aune et al. [33]	RR varied by BMI, specified by sex	—	Aune et al. [33]	Normal distribution
Disease prevalence, incidence, and mortality	Based on sex and age‐specific prevalence, incidence, and mortality data, specified by BMI and divided over the different health states in the model with RRs^b^	—	Different sources [29, 30, 31, 32, 33, 34, 35, 36, 37, 38]	Based on sex and age‐specific prevalence, incidence, and mortality data, specified by BMI and divided over the different health states in the model with RRs^b^	—	Different sources [29, 30, 31, 32, 33, 34, 35, 36, 37, 38]	Fixed
Discount rate:							
Costs	5%	0%	Agency for Health Technology Assessment and Tariff System [39]	3.50%	0%	NICE guideline [40]	—
Effects	3.50%	0%		3.50%	0%		—
Population:							
Proportion males, %	0.27	—	Percentage in trial	0.28	—	Percentage in trial	—
BMI, at start	32.27	—	Mean in trial	32.17	—	Mean in trial	—
Age, at start	43.61	—	Mean in trial	45.23	—	Mean in trial	—
**Effects**							
Intervention effect:							
PP+B vs. Control: Effect BMI, kg/m^2^ (SE)	−0.20 (0.34)	−0.86, 0.45	Trial results	−0.80 (0.41)	−1.60, 0.00	Trial results	Normal distribution
PP vs. Control: Effect BMI, kg/m^2^ (SE)	0.20 (0.33)	−0.45, 0.85	Trial results	−0.33 (0.41)	−1.14, 0.48	Trial results	
Effect loss BMI per year, percentage (proportion)	17.9% (0.1786)^c^	+/− 20%	Knowler et al. & Unick et al. [41, 42]	17.9% (0.1786)^c^	+/− 20%	Knowler et al. & Unick et al. [41, 42]	—
Duration effect loss BMI, years	5	1–7 years	Assumption based on Knowler et al. [41]	5	1–7 years	Assumption based on Knowler et al. [41]	
Intervention effect:							Normal distribution
PP+B vs. Control: Effect QoL, EQ‐5D‐5L utilities (SE)	−0.01 (0.01)	−0.04, 0.02	Trial results	0.03 (0.05)	−0.07, 0.13	Trial results	
PP vs. Control: Effect QoL, EQ‐5D‐5L utilities (SE)	0.00 (0.01)	−0.03, 0.03	Trial results	0.08 (0.05)	−0.02, 0.17	Trial results	
Duration effect QoL (intervention period), years	0.33	0.33–10 years	Trial duration [16]	0.33	0.33–10 years	Trial duration [15]	—
QALY—utility decrements for obesity‐related diseases:							
Diabetes	−0.067	—	Golicki et al. [43] and Sullivan et al. [44]	−0.0714	—	Heijink et al. [45] and Sullivan et al. [44]	Fixed
IHD	−0.059	—		−0.0626	—		Fixed
Stroke	−0.111	—		−0.1171	—		Fixed
**Costs 2020 £ (PLN)**							
Total intervention costs:							
PP+B:	546 (2733)	+/− 20%	Trial data	1175	+/− 20%	Trial data	Normal distribution
PP:	528 (2643)	+/− 20%	Trial data	1157	+/− 20%	Trial data	
Control:	274 (1369)	+/− 20%	Trial data	718	+/− 20%	Trial data	
Treatment of diseases:							
Diabetes	905 (4523)	+/− 20%	Lesniowska et al. [46]	1,584	+/− 20%	Hex et al. [47]	Gamma
IHD first year	10,700 (53,500)	+/− 20%	Palmer et al. [48]	6,756	+/− 20%	Alva et al. [49]	Gamma
IHD subsequent year	2190 (10,953)	+/− 20%	Palmer et al. [48]	4,280	+/− 20%	Alva et al. [49]	Gamma
Stroke first year	10,557 (52,785)	+/− 20%	Palmer et al. [48]	10,044	+/− 20%	Alva et al. [49]	Gamma
Stroke subsequent year	3772 (18,860)	+/− 20%	Palmer et al. [48]	5,282	+/− 20%	Alva et al. [49]	Gamma
Productivity loss:							
Hourly rate:	9.79 (48.7)	+/− 20%	Eurostat [50]	25.78	+/− 20%	Eurostat [50]	Gamma
Informal care:							
Hourly rate:	3.70 (18.5)	+/− 20%	Ecorys [51]	20.26	+/− 20%	Weatherly et al. [52]	Gamma
Unrelated medical costs	Depending on age and sex (+ divided by ‘last year of life’ and ‘other years of life’)	+/− 20%	Different sources (see Supporting Information S1)	Depending on age and sex (+ divided by ‘last year of life’ and ‘other years of life’)	+/− 20%	Different sources (see Hoogendoorn et al. [5])	Gamma
Non‐medical costs	Depending on age and sex	+/− 20%	Different sources [53, 54, 55]	Depending on age and sex	+/− 20%	Different sources (see Hoogendoorn et al. [5])	Gamma

Abbreviations: BMI, body mass index; CI, confidence interval; EQ‐5D, EuroQol five‐dimension questionnaire; IHD, ischemic heart disease; kg, kilogram; m, metre; PLN, Zloty; PP, personalised plan; PP+B, personalised plan + behavioural change; QALY, quality‐adjusted life years; QoL, quality of life; RR, relative risk; SE, standard error; UK, United Kingdom.

^a^ If studies did not specify whether data (i.e., the model inputs) pertained to Type 1 or Type 2 diabetes, it was assumed to be for Type 2 diabetes. Additionally, when data was provided for both Type 1 and Type 2 diabetes together, the combined data was utilised as model input for our analyses.

^b^ See Hoogendoorn et al. [5] for detailed methodology regarding calculation of transition probabilities. The same methods/sources were applied to both the UK and Poland, except for the overall case‐fatality rates for stroke in Poland, which were sourced from OECD, 2019 [56] instead of OECD, 2017 [38] as used by Hoogendoorn et al. [5] This change was made due to the lack of Polish data in the OECD report of 2017.

^c^ Knowler et al. [41] observed over a 5‐year period that participants in the study experienced an annual percentage of decreasing effect in weight loss (i.e., weight regain), starting with 100% weight loss (approximately 7 kg) in the first year, followed by a gradual gain in weight, resulting in 28.5% of weight loss (approximately 2 kg) from the initial 7 kg at the beginning of year 5. This translates into an average annual decrease of 17.86% in weight loss.

Transition probabilities varied according to age, sex, BMI, and health state and required data from different sources [5]. The Global Burden of Disease (GBD) study was used to derive mean BMI by age and sex in Poland and the UK [29]. A meta‐analysis of 230 cohort studies [33] yielded sex‐specific relative risks for the association between BMI and all‐cause mortality. Age‐specific relative risks for the association between BMI and diabetes, IHD, and stroke were derived from the GBD study [29]. Moreover, the relative risks for the co‐occurrence of diseases were considered [30, 31, 32]. Prevalence and incidence data for diabetes, IHD, and stroke, stratified by sex and age, were obtained from the DYNAMO‐HIA study, along with mortality data [31, 32]. Additional studies [34, 35, 36] and OECD data [37, 38] were used to calculate mortality, as explained in more detail elsewhere [5].

The trial data on BMI and health‐related quality of life (HRQoL) were used as effectiveness parameters for the interventions over the 4‐month trial period and extrapolated over a lifetime horizon. Differences in BMI effects between the intervention and control groups were calculated using linear mixed models, considering random intercepts and fixed effects [57]. Changes in BMI effects beyond the trial period were estimated using data from a longitudinal study [41]. Initially, this estimation involved no decrease in the measured BMI effect (from the trial) in the first year, indicating an ongoing effect of the intervention on BMI. Subsequently, a yearly effect reduction of 17.9% was assumed, reflecting weight regain, until the start of year 5. After this period, no further degradation in effect (i.e., no additional weight regain) was assumed. See footnotes in Table 1 for more explanation [41, 42].

HRQoL was measured using the EuroQol five‐dimension questionnaire with five levels (EQ‐5D‐5L), and an EQ‐5D index score (i.e., utilities) was calculated using country‐specific value sets [58, 59, 60]. Differences in utilities between the interventions (PP+B and PP) and control groups were analysed using generalised estimation equations [61, 62]. Long‐term health outcomes were expressed in life expectancy and quality‐adjusted life years (QALYs) as recommended in country‐specific guidelines for health technology assessments (HTAs) [39, 40, 63]. HRQoL within the model incorporated sex‐ and age‐specific utilities from the general population in Poland [43] and the UK [45], adjusted for the occurrence of diabetes, IHD, and stroke using prevalence data and established utility decrements for these diseases [44].

The base‐case analysis considered costs from a societal perspective, including intervention costs, medical costs (i.e., costs for treating obesity‐related diseases and [future] costs for other diseases [i.e., unrelated medical costs]), productivity costs, informal care costs, and non‐medical costs. Intervention costs were determined based on interviews and information provided by (PREVENTOMICS) project partners and participants and were based on a hypothetical scenario wherein the intervention would be introduced to the market. See Table 2 for the intervention cost components.

**Table 2:** Average intervention costs per participant in (A) Poland (2020 £ [PLN]) and (B) the UK (2020 £).

**A**					
Components	PP+B	PP	Control	Difference PP+B‐control	Difference PP‐control
1. Access for participant and centre (professional) + maintenance MetaDieta app/software^a^	27 (134)	27 (134)	0	27 (134)	27 (134)
2. Dietician/Nutritionist appointments^b^	100 (500)	100 (500)	100 (500)	0	0
3. Behavioural messages via app per participant	8.9 (45)	0	0	8.9 (45)	0
4. Behavioural message via app integration with MetaDieta + maintenance^c^	8.9 (45)	0	0	8.9 (45)	0
5. PREVENTOMICS platform (storage data + questionnaires maintenance)^d^	1.25 (6.23)	1.25 (6.23)	0	1.25 (6.23)	1.25 (6.23)
6. Extra costs grocery shopping (eating healthier)^e^	174 (869)	174 (869)	174 (869)	0	0
7. Tests (blood, urine, saliva)					
Omics	137 (687)	137 (687)	0	137 (687)	137 (687)
Genetics	48 (241)	48 (241)	0	48 (241)	48 (241)
Other (e.g., overhead)	41 (206)	41 (206)	0	41 (206)	41 (206)
**Total costs**	**546 (2733)**	**528 (2643)**	**274 (1369)**	**272 (1364)**	**254 (1274)**
Abbreviations: PLN, Zloty; PP, personalised plan; PP+B, personalised plan + behavioural change. ^**a**^A fixed amount of £2671 per year was charged. These costs were divided over 100 participants, since it was assumed that 100 participants will be the minimum treated persons by the dieticians per year. ^**b**^Unit costs per dietician appointment were multiplied by the number of sessions (*n* = 4) over the trial period. ^**c**^A fixed amount of £890 per year was charged. These costs were distributed over 100 participants, since it was assumed that 100 participants will be the minimum number of persons treated by the dieticians per year. ^**d**^A fixed amount of £125 per month was charged. These costs were divided over the total number of users of the platform, which equalled the total number of participants in all interventions in the PREVENTOMICS project (*n* = 400). ^**e**^Additional costs for grocery shopping due to healthier eating compared to peoples’ standard eating pattern were obtained from participants in the Polish trial.					

**B**					
Components	PP+B	PP	Control	Difference PP+B‐control	Difference PP‐control
1. Access for participant and centre (professional) + maintenance MetaDieta app/software^a^	27	27	0	27	27
2. Dietician/Nutritionist appointments^b^	383	383	383	0	0
3. Behavioural messages via app per participant	8.9	0	0	8.9	0
4. Behavioural message via app integration with MetaDieta + maintenance^c^	8.9	0	0	8.9	0
5. PREVENTOMICS platform (storage data + questionnaires maintenance)^d^	1.25	1.25	0	1.25	1.25
6. Extra costs grocery shopping (eating healthier)^e^	335	335	335	0	0
7. Tests (blood, urine, saliva)					
Omics	279	279	0	279	279
Genetics	48	48	0	48	48
Other (e.g., overhead)	84	84	0	84	84
**Total costs**	**1175**	**1157**	**718**	**457**	**439**
Abbreviations: PP, personalised plan; PP+B, personalised plan + behavioural change. ^**a**^A fixed amount of £2671 per year was charged. These costs were divided over 100 participants, since it was assumed that 100 participants will be the minimum treated persons by the dieticians per year. ^**b**^Unit costs per dietician appointment were multiplied by the number of sessions (*n* = 4) over the trial period. ^**c**^A fixed amount of £890 per year was charged. These costs were divided over 100 participants, since it was assumed that 100 participants will be the minimum treated persons by the dieticians per year. ^**d**^A fixed amount of £125 per month was charged. These costs were divided over the total number of users of the platform, which equalled the total number of participants in all interventions in the PREVENTOMICS project (*n* = 400). ^**e**^Additional costs for grocery shopping due to healthier eating compared to peoples’ standard eating pattern were obtained from participants in the Polish trial and converted to £ with the PPP.					

Direct medical costs for diabetes, IHD, and stroke were obtained from relevant studies [46, 47, 48, 49]. Productivity costs were estimated using SHARE (survey of health ageing and retirement in Europe) data [64] for central European countries, which were also deemed appropriate for calculations for Poland because of comparable (un)employment status with that of central European countries [65]. The friction cost method was used to calculate long‐term work loss costs, considering employment rates, mean working hours per week, and probability of unemployment. Additionally, production costs per hour were obtained from Eurostat [50]. Informal care costs were also based on SHARE data [64], accounting for the percentage of people receiving informal care and daily hours of care. Regression equations for eastern European countries for Poland and for central European countries for the UK were used for these estimations. See Hoogendoorn et al. [5] for more details about the methodologies applied.

Unrelated medical costs were calculated by subtracting costs per capita for diabetes, IHD, and stroke from annual per capita healthcare spending by sex and age. Supporting Information S1 and Hoogendoorn et al. [5] provide more details about this calculation for Poland and the UK, respectively. Age‐specific non‐medical costs were estimated with the mean consumption expenditure [53], which was adjusted for household size [54] and corrected for the probability of having more than one adult per household [55].

All costs were converted to 2020 currency using the consumer price index for Poland and the UK [66]. Following the ISPOR's guideline on good research practices [67], the costs were then converted to PLN using purchasing power parity (PPP) [68] and exchange rates [69], depending on the source. Finally, all costs were converted from 2020 PLN to 2020 British pounds (£) using the exchange rate of 1 PLN = £0.2 [69]. According to the guidelines for HTA, costs were discounted by 5% in Poland and 3.5% in the UK; effects were discounted by 3.5% in both countries [39, 40, 63].

### 2.5 Cost‐Effectiveness Analysis

The model outcomes analysed in this study included costs (with a breakdown by component), life years, life years with diabetes, cumulative incident cases of IHD and stroke, and QALYs for each intervention. The cost‐effectiveness was assessed using the incremental cost‐utility ratio (ICUR), calculated as the ratio of incremental costs to incremental QALYs, comparing PP+B and PP to the control intervention. The willingness‐to‐pay threshold (WTP) for gaining one QALY was set at three times the gross domestic product (GDP) in 2020 per capita in Poland (approximately £34,000 [171,000 PLN] per QALY) [70, 71, 72] and at £20,000 per QALY in the UK [40].

### 2.6 Sensitivity Analyses

In the early stages of development, uncertainties are inevitable, necessitating sensitivity analyses within these early cost‐effectiveness analyses to address them [22, 73]. Univariate sensitivity analyses were conducted to test the impact of model assumptions and input parameters on the results. Each parameter was varied individually using the lower and upper limits of the 95% confidence interval (CI) or by +/−20% when the CI was unavailable. The intervention costs were adjusted by +/−20% to account for uncertainties in factors such as the number of people receiving the intervention. The results were presented using tornado diagrams.

Scenario analyses were conducted to examine cost‐effectiveness results for females and males separately. Moreover, since guidelines recommend excluding unrelated medical and non‐medical costs [40, 74], these costs were excluded in a scenario analysis as well. Another scenario included only healthcare‐related costs (healthcare perspective), as guidelines of HTA in Poland and the UK recommend using a payer perspective on costs [39, 40, 63]. Additionally, undiscounted results were presented to demonstrate the impact of discount rates. Lastly, a scenario analysis was conducted by comparing PP+B and PP with ‘no intervention’. Notably, for the ‘no intervention’ arm, we assumed no measured effect, emphasising a comparison to people's current eating habits.

PSAs were conducted with 5000 iterations to examine the combined impact of uncertainties about the values of input parameters on the estimated cost‐effectiveness results. The PSAs included uncertainty in costs, relative risks associated with BMI and all‐cause mortality, and BMI and obesity‐related diseases. See Table 1 for more details. The results were presented in cost‐effectiveness planes and cost‐effectiveness acceptability curves (CEACs) [75]. Additional details on the PSA inputs can be found in Supporting Information S2.

## 3 Results

### 3.1 Trial Outcomes

In Poland, 89 participants were randomised to the PP+B group, 88 to the PP group and 88 to the control group. Baseline characteristics did not differ significantly among the groups in terms of age, sex, BMI, and HRQoL (see Supporting Information S3). There were 57 participants who completed the study in the PP+B group, 61 in the PP group and 52 in the control group. All groups showed a significant decrease in BMI, with the PP+B group having a slightly greater but nonsignificant decrease in BMI compared to the control group (i.e., difference of 0.20 kg/m^2^, 95% CI: −0.86, 0.45 kg/m^2^) and the PP group showing a slightly smaller but nonsignificant decrease in BMI compared to control (i.e., difference of 0.20 kg/m^2^, 95% CI: −0.45, 0.85 kg/m^2^). No significant differences were found in EQ‐5D utilities between groups. In the UK, 20 participants completed the study in the PP+B group, 19 in the PP group and 15 in the control group and were included in the analyses. All groups showed a decrease in BMI, which was significant in the PP+B and the PP groups, and both the PP+B and PP groups had a greater but nonsignificant decrease in BMI compared to the control group (PP+B: −0.80 kg/m^2^, 95% CI: −1.60, 0.00 kg/m^2^; PP: −0.33 kg/m^2^, 95% CI: −1.14, 0.48 kg/m^2^). Moreover, both the PP+B and PP groups had a greater but nonsignificant increase in EQ‐5D utilities compared to the control group (PP+ B: 0.03 utilities, 95% CI: −0.07, 0.13; PP: 0.08 utilities, 95% CI: −0.02, 0.17). More details can be found in Supporting Information S4.

In Poland, the intervention costs per participant were £546 (2733 PLN) in the PP + B group and £528 (2643 PLN) in the PP group. The control group had total costs per participant of £274 (1369 PLN), which included dietician appointments and extra grocery costs. In the UK, the intervention costs were £1175 in the PP + B group, £1157 in the PP group, and £718 in the control group. The differences in costs between PP+B or PP and the control groups can be attributed to the costs for testing. See Table 2 for more details.

### 3.2 Base‐Case Results

The trial‐based effects and costs were extrapolated over a lifetime horizon, leading to various health outcomes and costs summarised in Table 3. In Poland, PP+B slightly increased health by 0.011 QALYs with costs of £227 (1133 PLN) compared to control. This resulted in an ICUR of £20,404 (102,018 PLN) per QALY, indicating cost‐effectiveness (i.e., ICUR is below the WTP threshold of £34,000 [171,000 PLN]). The PP intervention slightly decreased health by 0.015 QALYs and increased costs by £302 (1509 PLN), making the control intervention dominant.

**Table 3:** Cost‐effectiveness analysis results (discounted).

Scenario	Strategy	Costs (£ [PLN])	Incremental costs (£ [PLN])	Effectiveness (in QALYs)	Incremental effectiveness (in QALYs)	ICUR (£ [PLN] per QALY)
**Poland**						
Base case (societal perspective)	PP+B	79,805 (399,025)	227 (1133)	16.536	0.011	20,404 (102,018)
	PP	79,880 (399,401)	302 (1509)	16.51	−0.015	Control dominates
	Control	79,578 (397,892)	—	16.525	—	—
Societal perspective without unrelated medical costs and non‐medical costs	PP+B	13,880 (69,399)	194 (971)	16.536	0.011	17,655 (88,273)
	PP	14,020 (70,102)	335 (1673)	16.510	−0.015	Control dominates
	Control	13,686 (68,429)	—	16.525	—	—
Healthcare perspective	PP+B	9440 (47,201)	195 (977)	16.536	0.011	17,764 (88,818)
	PP	9579 (47,893)	333 (1667)	16.510	−0.015	Control dominates
	Control	9245 (46,225)	—	16.525	—	—
Male	PP+B	73,072 (365,360)	84 (422)	15.81	0.126	669 (3343)
	PP	73,055 (365,276)	67 (337)	15.813	0.128	525 (2625)
	Control	72,988 (364,938)	—	15.684	—	—
Female	PP+B	82,331 (411,657)	383 (1915)	16.745	−0.031	Control dominates
	PP	82,417 (412,085)	469 (2343)	16.725	−0.05	Control dominates
	Control	81,948 (409,742)	—	16.775	—	—
**UK**						
Base case (societal perspective)	PP+B	299,438	576	16.023	0.044	13,006
	PP	299,351	489	16.019	0.04	12,222
	Control	298,862	—	15.979	—	—
Societal perspective without unrelated medical costs and non‐medical costs	PP+B	34,389	129	16.023	0.044	2932
	PP	34,563	301	16.019	0.04	7525
	Control	34,260	—	15.979	—	—
Healthcare perspective	PP+B	15,750	112	16.023	0.044	2545
	PP	15,934	294	16.019	0.04	7350
	Control	15,638	—	15.979	—	—
Male	PP+B	279,640	711	14.782	0.071	10,075
	PP	279,805	875	14.82	0.109	8017
	Control	278,930	—	14.711	—	—
Female	PP+B	307,147	517	16.524	0.033	15,685
	PP	307,064	435	16.512	0.02	21,225
	Control	306,629	—	16.491	—	—

Abbreviations: PLN, Zloty; PP, personalised plan; PP+B, personalised plan + behavioural change; QALY, quality‐adjusted life years; UK, United Kingdom.

In the UK, both PP+B and PP interventions slightly increased health (0.044 and 0.04 QALYs, respectively) compared to control. Additionally, the interventions led to increased costs, which were £576 and £489 higher, respectively, for PP+B and PP compared to the control. The ICURs were £13,006 and £12,222 per QALY, respectively for PP+B and PP, and were below the WTP threshold of £20,000 per QALY, indicating cost‐effectiveness. Slightly lower ICURs were found in both countries and interventions when costs and effects were not discounted. See Supporting Information S5 for more details on health outcomes and costs (both discounted and undiscounted).

### 3.3 Sensitivity Analyses

The analyses were also conducted from two other cost perspectives (see Table 3). Excluding unrelated medical costs and non‐medical costs, which meant the use of a narrower type of societal perspective, led to a slightly lower ICUR in Poland of £17,655 (88,273 PLN) per QALY for the PP+B intervention. In the UK, ICURs decreased for PP+B and PP, with ICURs of £2932 and £7525 per QALY, respectively. Use of the healthcare perspective resulted in ICURs that were similar to the ones seen when the narrower societal perspective was used. When we compared PP+B and PP with ‘no intervention’, the analyses showed an increase in both incremental QALYs and costs in both countries and groups compared to the base‐case scenario. In the UK, the rise in incremental costs was greater than the increase in incremental QALYs, leading to less favourable cost‐effectiveness outcomes (i.e., higher ICERs) when compared to “no intervention” than when compared to the control. In contrast, in Poland, incremental QALYs increased more than costs, leading to more cost‐effective results (i.e., lower ICERs). See Supporting Information S6 for more details about these results.

Baseline characteristics (age and BMI) and trial outcomes (BMI and utilities) separated by sex were used to model health outcomes and costs for females and males separately. See Supporting Information S7 for the model inputs for these subgroup analyses. The ICURs for both interventions (PP + B and PP) were lower for males than females in both countries. This was primarily due to higher incremental effectiveness in males. See Table 3 for more details.

**Figure 2:**
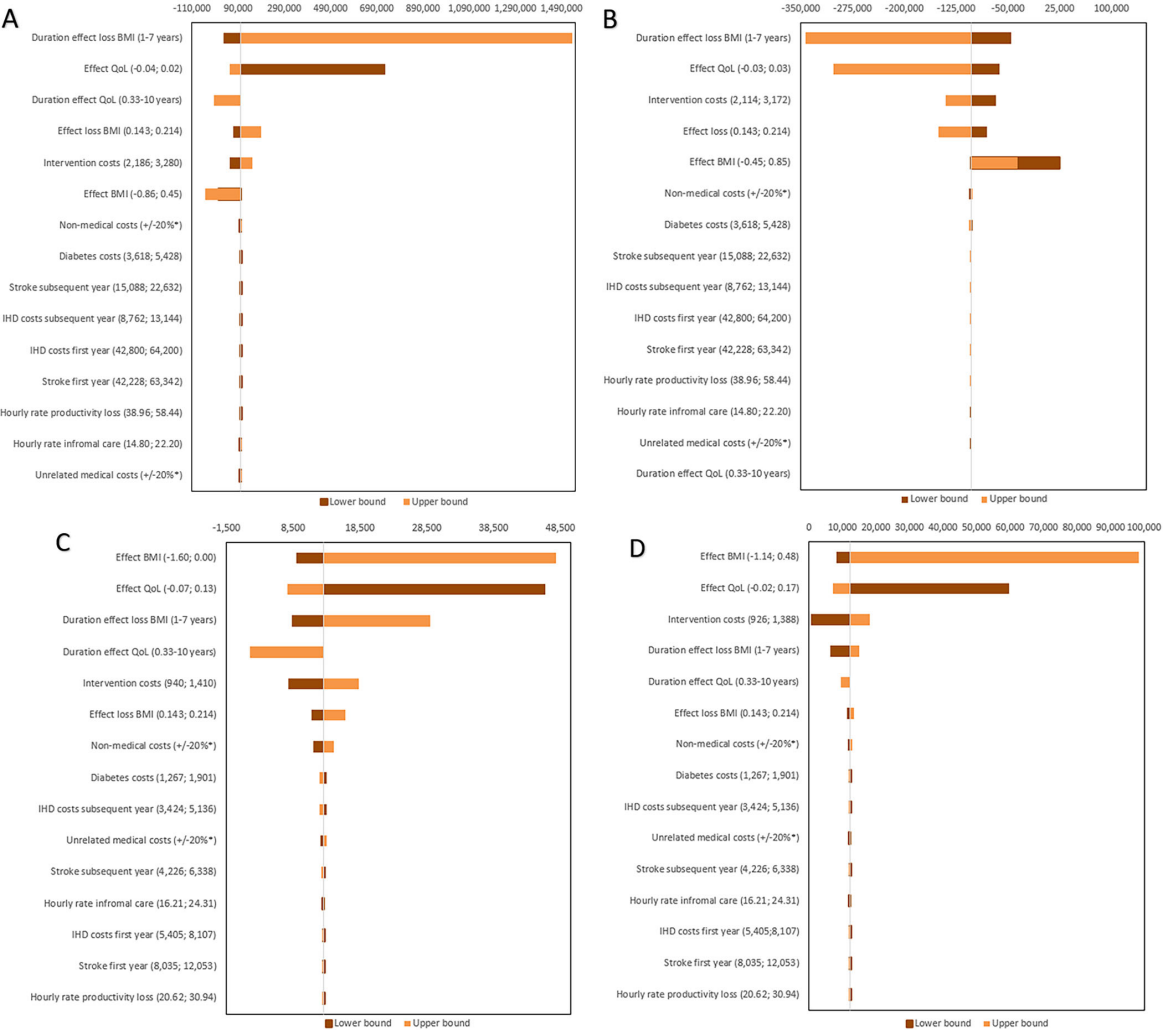
Tornado diagrams for change in ICURs using lower and upper bounds of parameters (A) for Poland in the PP+B group (in PLN per QALY), (B) for Poland in the PP group (in PLN per QALY), (C) for the UK in the PP+B group (in £ per QALY), and (D) for the UK in the PP group (in £ per QALY). BMI, body mass index; IHD, ischemic heart disease; QoL, quality of life. *No fixed number, since costs differ by sex and age.

Univariate sensitivity analyses in Poland revealed that the ICUR for both PP + B and PP interventions was most sensitive to changes in the duration of effect loss in BMI, resulting in an ICUR above the WTP threshold (i.e., £34,000 [171,000 PLN] per QALY) for the PP + B intervention (see Figure 2A,B). The second most influential parameter was the effectiveness in QoL, resulting in an ICUR above the WTP threshold (i.e., £34,000 [171,000 PLN] per QALY) for the PP+B intervention. When the lower bound of the effect in BMI was used in the PP intervention (i.e., the highest BMI loss of −0.45 kg/m^2^), an ICUR of £4784 (23,920 PLN) was found, which is below the WTP threshold and, therefore, indicates cost‐effectiveness. In the UK, the ICURs for both interventions were most sensitive to changes in intervention effectiveness on BMI and HRQoL, resulting in ICURs above the WTP threshold of £20,000 (see Figure 2C,D). See Supporting Information S8 for the tornado diagrams of the incremental QALYs and costs.

**Figure 3:**
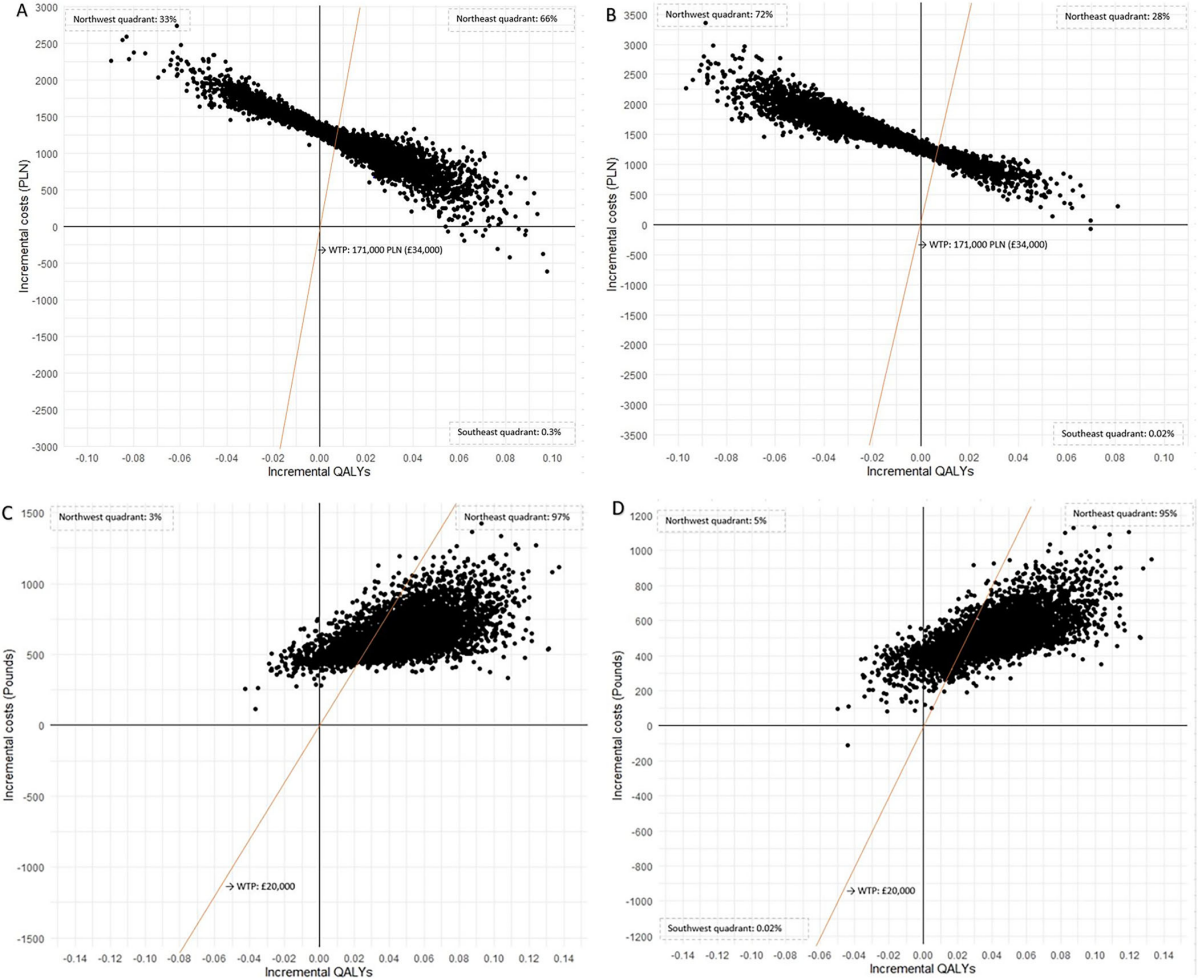
Cost‐effectiveness plane resulted from the probabilistic sensitivity analysis in (A) Poland: PP+B versus control, (B) Poland: PP versus control, (C) UK: PP+B versus control, and (D) UK: PP versus control. PLN, Zloty; QALYs, quality‐adjusted life years; WTP, willingness‐to‐pay.

Figure 3 shows the incremental cost‐effectiveness scatterplot for both interventions in both countries resulting from the PSA. For Poland, most values for the PP + B group are in the northeast quadrant, which indicates that there is a 66% chance that the PP + B intervention would increase costs and effectiveness. Moreover, most ICURs are below the WTP threshold (i.e., to the right of the diagonal line), indicating a 57% probability that PP + B is cost‐effective. This is reflected in Supporting Information S1: Figure S3 in Supporting Information S9, which shows the CEACs. For the PP interventions, there was only a 19% chance that PP is cost‐effective compared to the control intervention. In the UK, both interventions showed a very similar likelihood of being cost‐effective, with a probability of 75% to be cost‐effective for PP+B and 76% for PP.

## 4 Discussion

This study evaluated the potential cost‐effectiveness of two personalised nutrition interventions (PP+B and PP) compared to a control intervention in adults with overweight and obesity in Poland and the UK. The results from these ‘early’ cost‐effectiveness analyses can assist developers and policymakers when making decisions regarding future development, implementation and reimbursement [20, 21, 22]. The trials showed statistically nonsignificant differences in short‐term health outcomes (i.e., differences in BMI reduction) between the interventions and control. Moreover, the interventions led to higher costs. Extrapolated lifetime (base‐case) results showed in Poland that the PP+B intervention had an ICUR of £20,404 (102,018 PLN) per QALY, which is below the WTP threshold, while the PP intervention was dominated by the control. In the UK, both interventions resulted in ICURs below the WTP threshold (PP+B: £13,006 per QALY; PP: £12,222 per QALY). These findings suggest that personalised nutrition interventions can potentially be cost‐effective in promoting health and managing weight.

However, wide uncertainty was found (reflected in wide 95% CIs) surrounding the estimated short‐term effectiveness of the personalised nutrition interventions. Given this wide uncertainty, it is important to examine the likelihood that these interventions could be (cost)‐effective. This was done through PSAs, which revealed that there is a high probability of cost‐effectiveness for both PP+B (75%) and PP (76%) interventions in the UK. In Poland, the probability of cost‐effectiveness was 57% for PP+B and 19% for PP. Our results align well with the findings of a recent systematic review of cost‐effectiveness studies in personalised nutrition [76]. The review reported approximately 55% of the studied interventions to be cost‐effective at a threshold of $20,000 (£15,700) and 75% at a threshold of $55,000 (£43,000), which closely corresponds to the percentages we observed at the respective thresholds in the UK (£20,000) and Poland (£34,000; 171,000 PLN).

To account for uncertainties regarding the allocation of costs for personalised nutrition interventions, scenario analyses were conducted to explore how much the use of different cost perspectives affected the results. ICURs became even more favourable in the UK and for PP+B in Poland when unrelated medical costs and non‐medical costs were excluded, or when costs were considered from healthcare perspectives.

While uncertainties remain, these findings highlight the potential of personalised nutrition. Although widespread implementation may still be premature, policymakers should start exploring reimbursement strategies to ensure timely access once there is sufficient evidence to justify reimbursement. Unlike pharmaceutical products, interventions like PREVENTOMICS are typically not reimbursed at the national level in Poland and the UK [77, 78, 79, 80]. However, making (cost‐)effective personalised nutrition interventions accessible is crucial, particularly for lower socioeconomic groups, who face a higher risk of obesity [81], and may struggle to afford these interventions. Without fair financing, health inequalities could widen. Therefore, it is important to consider sustainable funding options at this early stage.

To assess the lifetime cost‐effectiveness of personalised nutrition interventions, various factors, particularly those related to ‘weight loss,’ had to be carefully considered. In our modelling study, we used trial results for BMI as a proxy for ‘weight loss’. Alternative outcome measures such as waist circumference or body fat have been proposed, because of its metabolic implications [1, 82]. Moreover, additional outcomes related to carbohydrate and lipid metabolism, which reduce the risk of diseases such as diabetes and atherosclerosis, could have been considered in the analyses, providing insights into the independent benefits of dietary management beyond changes in body weight [83]. However, we opted for BMI for two reasons. First, a validated economic model that examined the cost‐effectiveness of the interventions used BMI as a continuous parameter, which provided flexibility in simulating personalised nutrition's impact [5]. Second, there was insufficient data in the literature to support the use of other outcomes measures like waist circumference or body fat in the model to calculate the lifetime (cost‐)effectiveness. Future research should explore other relevant health outcomes, such as (more precise) estimations of relative risks concerning the association between waist circumference and all‐cause mortality or obesity‐related diseases. This data can subsequently be used to model the cost‐effectiveness of personalised nutrition using other health outcomes (e.g., waist circumference).

Additionally, future research should carefully consider the choice of comparator, as it significantly impacts cost‐effectiveness outcomes. This was evident in our scenario analysis, where comparing the interventions to ‘no intervention’ yielded different results. In this study, we believe the selected comparator was appropriate for our cost‐effectiveness analyses, as it isolates the effect of personalisation (in PP vs. control) and the behavioural change component (in PP+B vs. control). Future studies may use alternative comparators to address different research questions.

Besides, another scenario analysis highlighted potential differences in cost‐effectiveness between males and females. This aligns with previous studies suggesting that males tend to respond more positively to dietary interventions than females [84, 85]. However, given the uncertainty about the findings, future research should explore whether these results can be generalised from trial populations to the broader eligible population. Moreover, it is important for future studies to highlight all ethical issues related to personalised nutrition interventions, for example by conducting a comprehensive HTA, where interventions are systematically evaluated and assessed in the context of clinical, ethical, economic, social, legislative, organisational, and other domains [86].

This study has several limitations. First, while the model used in the cost‐effectiveness analyses offers valuable benefits, it also has its constraints. Only three main obesity‐related diseases were modelled directly, meaning the impact of BMI reduction on other conditions—such as osteoarthritis, sleep apnea, various cancers, and mental illness [87, 88, 89]—was not explicitly captured. However, their effect on life expectancy was considered through BMI‐related relative risks for all‐cause mortality, and associated costs were included in the unrelated medical costs. For a detailed discussion of the model and its limitations, see Hoogendoorn et al. [5].

Second, although short‐term effectiveness data were based on appropriately designed and executed clinical trials, the trial populations were relatively small (i.e., might be underpowered to detect differences between groups in BMI effect), leading to limited statistical power and uncertainty about effectiveness. However, as previously demonstrated in the literature [22, 73], uncertainties are inherent in early cost‐effectiveness analyses. To mitigate these uncertainties, sensitivity analyses were conducted. These analyses demonstrated, for example, that using the lower confidence limit of BMI effectiveness (i.e., −0.45 kg/m^2^) in Poland resulted in the possibility that the PP intervention is cost‐effective (ICUR: £4784 (23,920 PLN) per QALY). Larger trials with more participants are needed to obtain more precise estimates of effectiveness.

Third, the trials were likely too short to capture all relevant health effects, including long‐term benefits and sustained behavioural changes. This may have particularly affected the assessment of the behavioural change component in PP+B. In Poland, adding this component led to more favourable cost‐effectiveness outcomes compared to the PP group. However, the expected pattern was not observed in the UK. One potential explanation for this discrepancy is that while the behavioural change component led to a greater reduction in BMI in the UK, another key factor influencing cost‐effectiveness—the HRQoL measured by EQ‐5D utilities—showed a different trend. Specifically, the increase in EQ‐5D utilities was larger in the PP group compared to the control, whereas this effect was smaller in the PP+B group. As a result, the overall cost‐effectiveness outcomes of the two intervention groups remained similar, despite the greater BMI reduction in PP+B. Additionally, the literature suggests that the most significant improvements from nutrition interventions often emerge after 6 months [90]. Habit formation related to eating, drinking, or physical activity can take anywhere from 18 to 254 days, with an average of 66 days to establish automaticity [91]. It is possible that neither the full impact of personalised nutrition nor the behavioural change component was fully captured during the trial, given its 4‐month duration. Future trials should, therefore, include longer follow‐up periods to assess the sustainability of behavioural changes and better inform model assumptions.

Lastly, we used different protocols for analysing trial data in the UK and Poland, to ensure compatibility with the protocol used by the trialists in the two countries, which resulted in an intention‐to‐treat approach in Poland but a per‐protocol approach in the UK. However, the use of different protocols is not concerning for two reasons. First, changing the protocol would not have affected the estimated cost‐effectiveness. For example, use of an intention‐to‐treat protocol in the UK would have meant including six participants; adding them led to similar estimates of clinical outcomes (i.e., changes in BMI) and cost‐effectiveness. Second, we were not specifically aiming to compare cost‐effectiveness between countries.

## 5 Conclusions

This study provides evidence on the cost‐effectiveness of personalised nutrition interventions studied in Poland and the UK as part of the PREVENTOMICS project. Both PP+B and PP interventions in the UK demonstrated that they may be able to improve health outcomes slightly at acceptable extra costs, making them potentially cost‐effective compared to the control intervention. In Poland, results from the base‐case analysis showed that PP+B may be cost‐effective. Sensitivity analyses showed potential (better) cost‐effectiveness outcomes by using CIs of trial effectiveness. Larger and longer trials are recommended to further validate the results and to measure long‐term behavioural changes.

## Author Contributions

All authors contributed to the study conception and design. Material preparation, data collection and analysis were performed by Milanne Maria Johanna Galekop and William Ken Redekop. The first draft of the manuscript was written by Milanne Maria Johanna Galekop and Milanne Maria Johanna Galekop, Philip C. Calder, Ella J. Baker, Joanna Góralska, Urszula Raźny, Malgorzata Malczewska‐Malec, Carin A. Uyl‐de Groot and William Ken Redekop commented on subsequent versions of the manuscript. All authors read and approved the final version of the manuscript.

## Ethics Statement

The authors have nothing to report.

## Conflicts of Interest

At the time of submission, Milanne M.J. Galekop was an employee at GlaxoSmithKline BV (GSK), but GSK was not involved in this study in any way nor did this author contribute to this study in the capacity of her role within GSK. The other authors declare no conflicts of interest.

### 1 Peer Review

The peer review history for this article is available at https://www.webofscience.com/api/gateway/wos/peer-review/10.1111/jhn.70071.

## Supporting information

Supporting information final revision final.

## Data Availability

The data that support the findings of this study are available from the corresponding author upon reasonable request.

## References

[1] World Health Organization , Factsheet, Obesity and Overweight, https://www.who.int/news-room/fact-sheets/detail/obesity-and-overweight. Published 2021x, accessed January 18, 2022.

[2] OECD , The Heavy Burden of Obesity: The Economics of Prevention, 10.1787/67450d67-en. Published 2019, accessed January 18, 2022.

[3] G. P. Wulandari and S. A. Kristina , “Direct and Indirect Cost of Obesity: A Systematic Review,” Global Journal of Health Science 10, no. 9 (2018): 122–131.

[4] A. Dee , K. Kearns , C. O'Neill , et al., “The Direct and Indirect Costs of Both Overweight and Obesity: A Systematic Review,” BMC Research Notes 7, no. 1 (2014): 242. 24739239 10.1186/1756-0500-7-242 PMC4006977

[5] M. Hoogendoorn , M. M. J. Galekop , and P. van Baal, , “The Lifetime Health and Economic Burden of Obesity in Five European Countries: What Is the Potential Impact of Prevention?,” Diabetes, Obesity & Metabolism 25, no. 8 (2023): 2351–2361. 10.1111/dom.15116 37222003

[6] L. Qi , “Personalized Nutrition and Obesity,” Annals of Medicine 46, no. 5 (2014): 247–252. 24716734 10.3109/07853890.2014.891802 PMC5330214

[7] J. M. Ordovas and D. Corella , “Nutritional Genomics,” Annual Review of Genomics and Human Genetics 5 (2004): 71–118. 10.1146/annurev.genom.5.061903.180008 15485344

[8] R. Fallaize , A. L. Macready , L. T. Butler , et al., “The Perceived Impact of The National Health Service on Personalised Nutrition Service Delivery Among the UK Public,” British Journal of Nutrition 113, no. 8 (2015): 1271–1279. 25812432 10.1017/S0007114515000045 PMC4416278

[9] J. M. Ordovas , L. R. Ferguson , E. S. Tai , and J. C. Mathers , “Personalised Nutrition and Health,” BMJ (Online) 361 (2018): 1–7. 10.1136/bmj.k2173 PMC6081996 29898881

[10] P. Perez‐Martinez , M. C. Phillips , J. Delgado‐Lista , A. Garcia‐Rios , J. Lopez‐Miranda , and F. Perez‐Jimenez , “Nutrigenetics, Metabolic Syndrome Risk and Personalized Nutrition,” Current Vascular Pharmacology 11, no. 6 (2013): 946–953. 24168447 10.2174/157016111106140128120911

[11] J. Lopez‐Miranda , C. Williams , and D. Lairon , “Dietary, Physiological, Genetic and Pathological Influences on Postprandial Lipid Metabolism,” British Journal of Nutrition 98, no. 3 (2007): 458–473. 17705891 10.1017/S000711450774268X

[12] S. Shyam , K. X. Lee , A. S. W. Tan , et al., “Effect of Personalized Nutrition on Dietary, Physical Activity, and Health Outcomes: A Systematic Review of Randomized Trials,” Nutrients 14, no. 19 (2022): 4104. 36235756 10.3390/nu14194104 PMC9570623

[13] J. Keijer , X. Escoté , S. Galmés , et al., “Omics Biomarkers and an Approach for Their Practical Implementation to Delineate Health Status for Personalized Nutrition Strategies,” Critical Reviews in Food Science and Nutrition 19 (2023): 1–29. 10.1080/10408398.2023.2198605 37077157

[14] PREVENTOMICS, PREVENTOMICS Project, https://preventomics.eu/. Published 2022, accessed January 10, 2022.

[15] P. Calder, ISRCTN51509551: Personalised Advice to Aid Weight Loss, https://www.isrctn.com/ISRCTN51509551?q=personalised nutrition advice&filters=&sort=&offset=9&totalResults=22&page=1&pageSize=10. Published 2023, accessed May 17, 2023.

[16] M. Malczewska‐Malec, ISRCTN46063864: Personalised Nutritional Advice to aid Weight Loss, https://www.isrctn.com/ISRCTN46063864?q=preventomics&filters=&sort=&offset=1&totalResults=1&page=1&pageSize=10. Published 2023, accessed May 17, 2023.

[17] K. H. Cerri , M. Knapp , and J. L. Fernandez , “Decision Making by NICE: Examining the Influences of Evidence, Process and Context,” Health Economics, Policy and Law 9, no. 2 (2014): 119–141. 23688554 10.1017/S1744133113000030

[18] D. D. Wang and F. B. Hu , “Precision Nutrition for Prevention and Management of Type 2 Diabetes,” Lancet Diabetes & Endocrinology 6, no. 5 (2018): 416–426. 29433995 10.1016/S2213-8587(18)30037-8

[19] O. Ramos‐Lopez , J. A. Martinez , and F. I. Milagro , “Holistic Integration of Omics Tools for Precision Nutrition in Health and Disease,” Nutrients 14, no. 19 (2022): 4074. 36235725 10.3390/nu14194074 PMC9572439

[20] J. Love‐Koh , “How Useful Are Early Economic Models? Comment on ‘Problems and Promises of Health Technologies: The Role of Early Health Economic Modelling.ʼ,” International Journal of Health Policy and Management 9, no. 5 (2020): 215–217. 32563224 10.15171/ijhpm.2019.119 PMC7306112

[21] M. J. IJzerman and L. M. G. Steuten , “Early Assessment of Medical Technologies to Inform Product Development and Market Access: A Review of Methods and Applications,” Applied Health Economics and Health Policy 9, no. 5 (2011): 331–347. 21875163 10.2165/11593380-000000000-00000

[22] M. J. IJzerman , H. Koffijberg , E. Fenwick , and M. Krahn , “Emerging Use of Early Health Technology Assessment in Medical Product Development: A Scoping Review of the Literature,” PharmacoEconomics 35, no. 7 (2017): 727–740. 28432642 10.1007/s40273-017-0509-1 PMC5488152

[23] D. Husereau , M. Drummond , S. Petrou , et al., “Consolidated Health Economic Evaluation Reporting Standards (CHEERS) Statement,” BMJ (Clinical Research ed.) 346, no. March (2013): 1049. 10.1136/bmj.f1049 23529982

[24] International Diabetes Federation , The IDF Consensus Worldwide Definition of the Metabolic Syndrome. IDF Commun; 2006.

[25] V. Yumuk , C. Tsigos , M. Fried , et al., “European Guidelines for Obesity Management in Adults,” Obesity Facts 8, no. 6 (2015): 402–424. 26641646 10.1159/000442721 PMC5644856

[26] C. D'Antonio, PREVENTOMICS Final Conference: Software to Elaborate and Deliver a Personalised Diet, https://preventomics.eu/preventomics-final-conference/. Published 2022, accessed January 5, 2023.

[27] E. Bothos PREVENTOMICS Final Conference: Recommender System, Integration in Third‐Party Apps, https://preventomics.eu/preventomics-final-conference/. Published 2022, accessed January 5, 2023.

[28] S. van Berlo, PREVENTOMICS Final Conference: Do‐Omics, a Behavioural Change Programme, https://preventomics.eu/preventomics-final-conference/. Published 2022, accessed January 5, 2023.

[29] GBD 2015 Obesity Collaborators , “Health Effects of Overweight and Obesity in 195 Countries Over 25 Years,” New England Journal of Medicine 377, no. 1 (2017): 13–27. 28604169 10.1056/NEJMoa1614362 PMC5477817

[30] P. H. van Baal , P. M. Engelfriet , H. C. Boshuizen , J. van de Kassteele , F. G. Schellevis , and R. T. Hoogenveen , “Co‐Occurrence of Diabetes, Myocardial Infarction, Stroke, and Cancer: Quantifying Age Patterns in the Dutch Population Using Health Survey Data,” Population Health Metrics 9 (2011): 51. 21884614 10.1186/1478-7954-9-51 PMC3175448

[31] DYNAMO‐HIA Project, *Workpackage 7: Overweight and Obesity, Report on Data Collection for Overweight and Obesity Prevalence and Related Relative Risks*, https://www.dynamo-hia.eu/sites/default/files/2018-04/BMI_WP7-datareport_20100317.pdf. Published 2010, accessed March 12, 2020.

[32] H. C. Boshuizen , S. K. Lhachimi , P. H. M. van Baal , et al., “The DYNAMO‐HIA Model: An Efficient Implementation of a Risk Factor/Chronic Disease Markov Model for Use in Health Impact Assessment (HIA),” Demography 49, no. 4 (2012): 1259–1283. 23055232 10.1007/s13524-012-0122-z

[33] D. Aune , A. Sen , and M. Prasad , et al., “BMI and All Cause Mortality: Systematic Review and Non‐Linear Dose‐Response Meta‐Analysis of 230 Cohort Studies With 3.74 Million Deaths Among 30.3 Million Participants,” BMJ (Online) 353 (2016): i2156. 10.1136/bmj.i2156 PMC4856854 27146380

[34] R. T. Hoogenveen , H. C. Boshuizen , P. M. Engelfriet , and P. H. M. Van Baal , “You Only Die Once: Accounting for Multi‐Attributable Mortality Risks in Multi‐Disease Models for Health‐Economic Analyses,” Medical Decision Making 37, no. 4 (2017): 403–414. 27405746 10.1177/0272989X16658661

[35] I. Vaartjes , I. van Dis , D. E. Grobbee , and M. L. Bots , “The Dynamics of Mortality in Follow‐Up Time After an Acute Myocardial Infarction, Lower Extremity Arterial Disease and Ischemic Stroke,” BMC Cardiovascular Disorders 10 (2010): 57. 21106115 10.1186/1471-2261-10-57 PMC3003625

[36] I. Vaartjes , J. B. Reitsma , M. Berger‐Van Sijl , and M. L. Bots , “Gender Differences in Mortality After Hospital Admission for Stroke,” Cerebrovascular Diseases 28, no. 6 (2009): 564–571. 19844096 10.1159/000247600

[37] OECD, (2017), *Health at a Glance 2017: Mortality Following Acute Myocardial Infarction (AMI)*, 10.1787/health_glance-2017-34-en. Published 2017, accessed April 19, 2022.

[38] OECD, (2017), *Health at a Glance 2017: Mortality Following Ischaemic Stroke*, 10.1787/health_glance-2017-33-en. Published 2017, accessed April 19, 2022.

[39] Agency for Health Technology Assessment and Tariff System, Wytyczne Oceny Technologii Medycznych. Wyroby medyczne. Część I. Wyroby o zastosowaniu profilaktycznym u terapeutycznym, wersja 1.0. (Health Technology Assessment Guidelines. Medical Products. Part I. Devices for Prophylactic and Therapeutic Use, Version. 2021; (June): 1‐68.

[40] NICE, NICE Health Technology Evaluations: The Manual, https://www.nice.org.uk/process/pmg36/resources/nice-health-technology-evaluations-the-manual-pdf-72286779244741. Published 2022, accessed 18 March, 2023.

[41] W. C. Knowler , S. E. Fowler , R. F. Hamman , et al., “10‐Year Follow‐Up of Diabetes Incidence and Weight Loss in the Diabetes Prevention Program Outcomes Study,” Lancet (London, England) 374, no. 1 (2009): 1677–1686. 19878986 10.1016/S0140-6736(09)61457-4 PMC3135022

[42] J. L. Unick , D. Beavers , D. S. Bond , et al., “The Long‐Term Effectiveness of a Lifestyle Intervention in Severely Obese Individuals,” American Journal of Medicine 126, no. 3 (2013): 236–242.e2. 23410564 10.1016/j.amjmed.2012.10.010 PMC3574274

[43] D. Golicki and M. Niewada , “EQ‐5D‐5L Polish Population Norms,” Archives of Medical Science 1, no. 1 (2017): 191–200. 10.5114/aoms.2015.52126 PMC5206353 28144271

[44] P. W. Sullivan , J. F. Slejko , M. J. Sculpher , and V. Ghushchyan , “Catalogue of EQ‐5D Scores for the United Kingdom,” Medical Decision Making 31, no. 6 (2011): 800–804. 21422468 10.1177/0272989X11401031

[45] R. Heijink , P. Van Baal , M. Oppe , X. Koolman , and G. Westert , “Decomposing Cross‐Country Differences in Quality Adjusted Life Expectancy: The Impact of Value Sets,” Population Health Metrics 9, no. 1 (2011): 17. 21699675 10.1186/1478-7954-9-17 PMC3146826

[46] J. Leśniowska , A. Schubert , M. Wojna , I. Skrzekowska‐Baran , and M. Fedyna , “Costs of Diabetes and Its Complications in Poland,” European Journal of Health Economics 15, no. 6 (2014): 653–660. 10.1007/s10198-013-0513-0 PMC4059958 23820625

[47] N. Hex , C. Bartlett , D. Wright , M. Taylor , and D. Varley , “Estimating the Current and Future Costs of Type1 and Type2 Diabetes in the UK, Including Direct Health Costs and Indirect Societal and Productivity Costs,” Diabetic Medicine 29, no. 7 (2012): 855–862. 22537247 10.1111/j.1464-5491.2012.03698.x

[48] J. L. Palmer , G. Goodall , S. Nielsen , et al., “Cost‐Effectiveness of Insulin Aspart Versus Human Soluble Insulin in Type 2 Diabetes in Four European Countries: Subgroup Analyses From the PREDICTIVE Study,” Current Medical Research and Opinion 24, no. 5 (2008): 1417–1428. 18400145 10.1185/030079908x297295

[49] M. L. Alva , A. Gray , B. Mihaylova , J. Leal , and R. R. Holman , “The Impact of Diabetes‐Related Complications on Healthcare Costs: New Results From the UKPDS (UKPDS 84),” Diabetic Medicine 32, no. 4 (2015): 459–466. 25439048 10.1111/dme.12647

[50] Eurostat, Labour Cost Levels by NACE Rev, 2 Activity: LC_LCI_LEV, https://ec.europa.eu/eurostat/databrowser/view/LC_LCI_LEV__custom_709491/bookmark/table?lang=en&bookmarkId=eb78a6c4-aa9b-4210-ad86-6f9d003e1952. Published 2022, accessed March 10, 2022.

[51] Ecorys, *Study on Exploring the Incidence and Costs of Informal Long‐Term Care in the EU*, https://op.europa.eu/en/publication-detail/-/publication/bafbb918-2197-11ec-bd8e-01aa75ed71a1. Published 2021, accessed January 19, 2023.

[52] H. Weatherly , R. Faria , and B. Van Den Berg , “Valuing Informal Care for Economic Evaluation,” Encyclopedia of Health Economics 3 (2014): 459–467.

[53] Eurostat, Mean Consumption Expenditure by Age of the Reference Person: [hbs_exp_t135], https://appsso.eurostat.ec.europa.eu/nui/show.do?dataset=hbs_exp_t135&lang=en. Published 2021, accessed July 1, 2022.

[54] Eurostat, Household Characteristics by Age of the Reference Person: HBS_CAR_T314, https://ec.europa.eu/eurostat/databrowser/view/hbs_car_t314/default/table?lang=en. Published 2021, accessed July 1, 2022.

[55] K. Kellerborg , M. Perry‐Duxbury , L. de Vries , and P. van Baal , “Practical Guidance for Including Future Costs in Economic Evaluations in The Netherlands: Introducing and Applying PAID 3.0,” Value in Health 23, no. 11 (2020): 1453–1461. 33127016 10.1016/j.jval.2020.07.004

[56] OECD, *Health at a Glance 2019: Mortality Following Ischaemic Stroke*, 10.1787/4dd50c09-en. Published 2019, accessed January 5, 2023.

[57] J. D. Singer and J. B. Willet , Applied Longitudinal Data Analysis: Modeling Change and Event Occurrence (Oxford University Press, 2003).

[58] D. Golicki , M. Jakubczyk , K. Graczyk , and M. Niewada , “Valuation of EQ − 5D ‐ 5L Health States in Poland: The First EQ ‐ VT ‐ Based Study in Central and Eastern Europe,” Pharmaco Economics 37, no. 9 (2019): 1165–1176. 10.1007/s40273-019-00811-7 PMC6830402 31161586

[59] B. Van Hout , M. F. Janssen , Y. S. Feng , et al., “Interim Scoring for the EQ‐5D‐5L: Mapping the EQ‐5D‐5L to EQ‐5D‐3L Value Sets,” Value in Health 15, no. 5 (2012): 708–715. 22867780 10.1016/j.jval.2012.02.008

[60] NICE (National Institute for Health and Care Excellence), Position Statement on use of the EQ‐5D‐5L Value set for England (updated October 2019), https://www.nice.org.uk/about/what-we-do/our-programmes/nice-guidance/technology-appraisal-guidance/eq-5d-5l. Published 2022, accessed April 10, 2022.

[61] S. E. Wolowacz , A. Briggs , V. Belozeroff , et al., “Estimating Health‐State Utility for Economic Models in Clinical Studies: An ISPOR Good Research Practices Task Force Report,” Value in Health 19, no. 6 (2016): 704–719. 27712695 10.1016/j.jval.2016.06.001

[62] N. Devlin , D. Parkin , and B. Janssen , Methods for Analysing and Reporting EQ‐5D Data (Springer, 2020), 10.1007/978-3-030-47622-9. 33347096

[63] D. Sharma , A. K. Aggarwal , L. E. Downey , and S. Prinja , “National Healthcare Economic Evaluation Guidelines: A Cross‐Country Comparison,” PharmacoEconomics ‐ Open 5 (2021): 349–364. 33423205 10.1007/s41669-020-00250-7 PMC8333164

[64] A. Börsch‐Supan , M. Brandt , C. Hunkler , et al., “Data Resource Profile: The Survey of Health, Ageing and Retirement in Europe (Share),” International Journal of Epidemiology 42, no. 4 (2013): 992–1001. 23778574 10.1093/ije/dyt088 PMC3780997

[65] Eurostat, Unemployment by Sex and Age – Annual Data: UNE_RT_A, https://ec.europa.eu/eurostat/databrowser/view/UNE_RT_A__custom_2553687/bookmark/table?lang=en&bookmarkId=03a2bb70-40a8-4798-8682-d82d9c6701a0. Published 2022, accessed March 10, 2022.

[66] OECD, (2022), Inflation (CPI) (indicator), 10.1787/eee82e6e-en, accessed July 19, 2022.

[67] L. Shi , M. Hodges , M. Drummond , et al., “Good Research Practices for Measuring Drug Costs in Cost‐Effectiveness Analyses: An International Perspective: The ISPOR Drug Cost Task Force Report ‐ Part VI,” Value in Health 13, no. 1 (2010): 28–33. 19883403 10.1111/j.1524-4733.2009.00662.x

[68] OECD, (2022), Purchasing Power Parities (PPP) (Indicator), 10.1787/1290ee5a-en, accessed July 19, 2022.

[69] OECD, (2022), Exchange Rates (Indicator), 10.1787/037ed317-en, accessed July 19, 2022.

[70] M. Bertram , J. Lauer , K. De Joncheere , et al., “Cost‐Effectiveness Thresholds: Pros and Cons,” Bulletin of the World Health Organization 94, no. 12 (2016): 925–930. 27994285 10.2471/BLT.15.164418 PMC5153921

[71] O. Markiewicz , “Value of Life Year and Cost‐Effectiveness Thresholds: The Case of Poland,” Central European Economic journal 8, no. 55 (2021): 256–268.

[72] M. Niewada , B. Tabor , E. Piotrowicz , et al., “Cost‐Effectiveness of Telerehabilitation in Patients With Heart Failure in Poland: An Analysis Based on the Results of Telerehabilitation in the Heart Failure Patients (TELEREH‐HF) Randomized Clinical Trial,” Kardiologia Polska 79, no. 5 (2021): 510–516. 34125923 10.33963/KP.15885

[73] L. R. Buisman , M. P. M. H. Rutten‐Van Mölken , D. Postmus , J. J. Luime , C. A. Uyl‐De Groot , and W. K. Redekop , “The Early Bird Catches the Worm: Early Cost‐Effectiveness Analysis of New Medical Tests,” International Journal of Technology Assessment in Health Care 32, no. 1–2 (2016): 46–53. 27002226 10.1017/S0266462316000064

[74] B. Kearns, NICE DSU Report. The Relevance of Future, Unrelated Health Costs in Economic Evaluation in Nice Appraisals, http://nicedsu.org.uk/wp-content/uploads/2020/11/Future_unrelated_costs_Final.pdf. Published 2020, accessed January 19, 2023.

[75] A. Briggs , K. Claxton , and M. Sculper , Decision Modelling for Health Economic Evaluation (Oxford University Press, 2006).

[76] M. M. J. Galekop , C. A. Uyl‐de Groot , and W. Ken Redekop , “A Systematic Review of Cost‐Effectiveness Studies of Interventions With a Personalized Nutrition Component in Adults,” Value in Health 24, no. 3 (2021): 325–335. 33641765 10.1016/j.jval.2020.12.006

[77] M. J. Poley , “Nutrition and Health Technology Assessment: When Two Worlds Meet,” Frontiers in Pharmacology 6 (2015): 1–6. 26539116 10.3389/fphar.2015.00232 PMC4611967

[78] G. Kostelecki and A. Mastalerz‐Migas, Guidelines of the Polish Society of Dietetics and a National Consultant in the Field of Family Medicine Regarding Dietary Consultations as Part of Coordinated Care in Primary Health Care of January 31, 2023, https://www.gov.pl/attachment/2968553a-2aef-4d31-9ec3-c34a9d7844c7. Published 2023, accessed June 19, 2023.

[79] Minister of Health , Regulation of the Minister of Health (Rozporządzenie Ministra Zdrowia z Dnia), https://dziennikustaw.gov.pl/D2021000064201.pdf. Published 2022, accessed June 19, 2023.

[80] MedTech Europe , Recognising the Value of Digital Health Apps: An Assessment of Five European Healthcare Systems, https://www.medtecheurope.org/resource-library/recognising-the-value-of-digital-health-apps-an-assessment-of-five-european-healthcare-systems/. Published 2021, accessed June 18, 2023.

[81] A. Chatelan , M. Bochud , and K. L. Frohlich , “Precision Nutrition: Hype or Hope for Public Health Interventions to Reduce Obesity?,” International Journal of Epidemiology 48, no. 2 (2019): 332–342. 30544190 10.1093/ije/dyy274 PMC6469305

[82] R. A. Millstein , “Measuring Outcomes in Adult Weight Loss Studies That Include Diet and Physical Activity: A Systematic Review,” Journal of Nutrition and Metabolism 2014 (2014): 1–13. 10.1155/2014/421423 PMC4262752 25525513

[83] E. Hillesheim , X. Yin , G. P. Sundaramoorthy , and L. Brennan , “Using a Metabotype Framework to Deliver Personalized Nutrition Improves Dietary Quality and Metabolic Health Parameters: A 12‐Week Randomized Controlled Trial,” Molecular Nutrition & Food Research 67, no. 10 (2023): 1–12. 10.1002/mnfr.202200620 37038841

[84] L. M. Kent , D. P. Morton , P. M. Rankin , J. E. Gobble , and H. A. Diehl , “Gender Differences in Effectiveness of the Complete Health Improvement Program (CHIP),” Journal of Nutrition Education and Behavior 47, no. 1 (2015): 44–52. 25312267 10.1016/j.jneb.2014.08.016

[85] J. H. Shao and S. H. Chen , “Who Did It Better? Gender Differences in Effects of a Dietary Self‐Management Intervention for Older Community‐Dwelling Adults,” Journal of Women & Aging 33, no. 5 (2021): 473–486. 31880992 10.1080/08952841.2019.1707152

[86] B. O'Rourke , W. Oortwijn , and T. Schuller , “Announcing the New Definition of Health Technology Assessment,” Value in Health 23, no. 6 (2020): 824–825. 32540240 10.1016/j.jval.2020.05.001

[87] NHLBI , Managing Overweight and Obesity in Adults: Systematic Evidence Review From the Obesity Expert Panel, https://www.nhlbi.nih.gov/sites/default/files/media/docs/obesity-evidence-review.pdf. Published 2013, accessed December 18, 2024.

[88] National Heart Lung and Blood Institute , Clinical Guidelines on the Identification, Evaluation, and Treatment of Overweight and Obesity in Adults: The Evidence Report. Vol 98‐4083;1998.

[89] K. Bhaskaran , I. Douglas , H. Forbes , I. Dos‐Santos‐Silva , D. A. Leon , and L. Smeeth , “Body‐Mass Index and Risk of 22 Specific Cancers: A Population‐Based Cohort Study of 5·24 Million UK Adults,” The Lancet 384, no. 9945 (2014): 755–765. 10.1016/S0140-6736(14)60892-8 PMC4151483 25129328

[90] J. Jancey , A. H. Lee , A. P. James , et al., “Long‐Term Sustainability of a Physical Activity and Nutrition Intervention for Rural Adults With or at Risk of Metabolic Syndrome,” Australian and New Zealand Journal of Public Health 44, no. 5 (2020): 421–426. 32955747 10.1111/1753-6405.13036

[91] P. Lally , C. H. M. Van Jaarsveld , H. W. W. Potts , and J. Wardle , “How Are Habits Formed: Modelling Habit Formation in the Real World,” European Journal of Social Psychology 40 (2010): 998–1009.

