# Supplementary material for: Cost‐Effectiveness of Personalised Nutrition in Adults With Overweight and Obesity: PREVENTOMICS Studies in Poland and the UK
Source: J Hum Nutr Diet. 2025 Jun 2;38(3):e70071. doi: 10.1111/jhn.70071 (PMC12128005; doi:10.1111/jhn.70071)
Supplement: Supplementary file 1 — Supporting information final revision final. [file JHN-38-0-s001.docx]

# Supporting information

**Title**: Cost-effectiveness of personalised nutrition in adults with overweight and obesity: PREVENTOMICS studies in Poland and the UK

## Supplement 1: Method used to calculate unrelated costs.

Unrelated medical costs of Poland were calculated by subtracting the related costs per capita for diabetes, IHD and stroke from the annual health care spending per capita by sex and age. This annual health care spending per capita by sex and age was calculated following several steps.

1. Information from the DYNAMO-HIA project^1,2^ about the percentage of people with disability per age group, divided by sex (in year 2014), was used as a proxy for a certain distribution of the total health care costs over the Polish population.
2. The total health care expenditure of Poland in 2014,^3^ which was 107,458 PLN in millions of units, was divided over the different age groups by sex, based on the defined distribution in step 1. This enabled us to estimate a total expenditure per age group divided by sex.
3. The total health care expenditure per capita was calculated by dividing the total expenditure per age group divided by sex that was found in the second step, by the number of people in that age group divided by sex.^1,2^
4. The total health care expenditure per capita was divided by ‘last year of life’ and ‘other years of life’ with the following formula in R, using R Studio:

sc <- as,matrix(R_file_data_female$ac,tot)/(1 + (as,matrix(R_file_data_female$r,i)-1)*R_file_data_female$mr)

dc <- as,matrix(R_file_data_female$r,i)*sc

sc <- as,matrix(R_file_data_male$ac,tot)/(1 + (as,matrix(R_file_data_male$r,i)-1)*R_file_data_male$mr)

dc <- as,matrix(R_file_data_male$r,i)*sc

In this formula, ‘ac,tot’ is the same as total health care expenditure per capita as found in the third step, ‘r,i’ is the ratio of decedent/survivor as found in an article written by Kalseth et al. ^4^. It was assumed that this ratio could be applied in the Polish setting. ‘Mr’ is the overall mortality rate that was used from the DYNAMO_HIA study.^1,2^

This resulted in a total health care expenditure per capita divided by last year of life and other years of life.

1. Prevalence data of diabetes, IHD and stroke were obtained from the DYNAMO-HIA study,^1,2^ as well as incidence data of MI and stroke, all divided by age and sex.
2. The prevalence/incidence data together with the disease related costs per capita, were used to calculate the related costs. These costs were subtracted from the total health care expenditure costs per capita, to finally estimate the total unrelated health care costs per capita per age group divided by sex.
3. These costs were converted to 2020 PLN using the Consumer Price Index.^5^

## Supplement 2: Details on probabilistic sensitivity analyses from Hoogendoorn et al.^6^

To incorporate the inherent uncertainty in input parameters and its impact on model outcomes, a probabilistic sensitivity analysis (PSA) was conducted. The PSA encompassed uncertainty surrounding relative risks (RRs) associated with BMI and its relationship with all-cause mortality and obesity-related diseases, as well as cost uncertainties. Other parameters were held constant during the analysis.

For the RRs associated with BMI and all-cause mortality, uncertainty intervals of 11 different BMI values from Aune et al.^7^ were used. Ten random values were drawn from the intervals around the relative risks for each reported value of BMI, assuming a normal distribution. Using second-degree polynomial regression, a model was estimated with RR as the outcome and BMI as the predictor, based on the mean RRs and surrounding uncertainty. In the PSA, uncertainty was derived from the uncertainty around the coefficients in the estimated model and random draws for all coefficients were made by considering their covariance. Results were estimated separately for males and females.

A similar approach was used for RRs reflecting the association between BMI and obesity-related diseases. The Global Burden of Disease Study^8^ provided RRs for 12 age groups including their uncertainty intervals. Random draws (10 per observation) around each reported RR were taken and were assumed to follow a normal distribution. Subsequently, a linear model was estimated based on the reported RRs and surrounded uncertainty, using age as the predictor and RR as outcome. For the PSA, random draws for the coefficients were made while considering covariance.

Due to the absence of available data on cost uncertainty, a standard error (SE) of 20% of the mean value was assumed for all cost estimates. A gamma distribution was applied for cost uncertainty. The effectiveness estimates from the trial and intervention costs were assumed to follow a normal distribution.

## Supplement 3: Baseline characteristics participants PREVENTOMICS trials in Poland and the UK

**Table S1**: Baseline characteristics Poland^

|  | **PP+B, N= 89** | | | | | | **PP, N= 88** | | | | | | **Control, N= 88** | | | | | | **P-value** |  |
| --- | --- | --- | --- | --- | --- | --- | --- | --- | --- | --- | --- | --- | --- | --- | --- | --- | --- | --- | --- | --- |
|  |  |  |  |  |  |  |  |  |  |  |  |  |  |  |  |  |  |  |  |  |
|  | ***Mean*** | ***(sd)*** | ***Median*** | ***(IQR)*** | ***N*** | ***(%)*** | ***Mean*** | ***(sd)*** | ***Median*** | ***(IQR)*** | ***N*** | ***(%)*** | ***Mean*** | ***(sd)*** | ***Median*** | ***(IQR)*** | ***N*** | ***(%)*** |  |  |
| **Age, years** | 42 | 9.84 | 44 | (37, 48) |  |  | 44 | 10.96 | 45 | (35,51) |  |  | 45 | 10.83 | 47 | (38, 52) |  |  | 0.11 |  |
| **Sex** |  |  |  |  |  |  |  |  |  |  |  |  |  |  |  |  |  |  |  |  |
| Females |  |  |  |  | 63 | 71 |  |  |  |  | 69 | 78 |  |  |  |  | 61 | 69 | 0.35 |  |
| Males |  |  |  |  | 26 | 29 |  |  |  |  | 19 | 22 |  |  |  |  | 27 | 31 |  |  |
| **BMI (kg/m^2^)** | 31.82 | 4.78 | 31.25 | (27.99,35.08) |  |  | 32.75 | 4.53 | 32.53 | (29.06, 35.83) |  |  | 32.25 | 4.74 | 32.16 | (28.49, 35.82) |  |  | 0.29 |  |
| **EQ-5D-5L utility*** | 0.94 | 0.08 | 0.95 | (0.92, 0.97) |  |  | 0.91 | 0.10 | 0.94 | (0.90, 0.96) |  |  | 0.92 | 0.09 | 0.95 | (0.91, 0.98) |  |  | 0.15 |  |
| **EQ-5D VAS*** | 73.71 | 13.09 | 71 | (70,80) |  |  | 71.05 | 14.67 | 70 | (62.5, 80) |  |  | 71.08 | 17.25 | 75 | (60,82.5) |  |  | 0.72 |  |
| BMI, body mass index; EQ-5D, EuroQol five-dimension questionnaire; IQR, interquartile range; kg, kilogram; m, meter; n, number; PP, Personalised Plan; PP+B, Personalised plan + Behavioural change; sd, standard deviation; VAS, Visual Analogue Scale.  ^Sample size was calculated with the aim to detect a 2.40 cm difference in waist circumference between groups with 80% power with a two-tailed α of 0.05, to allow for a 20% drop out. | | | | | | | | | | | | | | | | | | | |  |
| **Only 88 respondents in the PP+B group filled in the questionnaire on HRQoL* | | | | | | | | | | | | | | | | | | | |  |
|  | | | | | | | | | | | | | | | | | | | |  |

**Table S2**: Baseline characteristics UK^

|  | **PP+B, N= 20** | | | | | | **PP, N= 15** | | | | | | **Control, N= 19** | | | | | | **P-value** |  |
| --- | --- | --- | --- | --- | --- | --- | --- | --- | --- | --- | --- | --- | --- | --- | --- | --- | --- | --- | --- | --- |
|  |  |  |  |  |  |  |  |  |  |  |  |  |  |  |  |  |  |  |  |  |
|  | ***Mean*** | ***(sd)*** | ***Median*** | ***(IQR)*** | ***N*** | ***(%)*** | ***Mean*** | ***(sd)*** | ***Median*** | ***(IQR)*** | ***N*** | ***(%)*** | ***Mean*** | ***(sd)*** | ***Median*** | ***(IQR)*** | ***N*** | ***(%)*** |  |  |
| **Age, years** | 45 | 12.14 | 48 | (35, 54) |  |  | 45 | 13.39 | 42 | (34, 56) |  |  | 46 | 14.26 | 44 | (36, 58) |  |  | 0.96 |  |
| **Sex** |  |  |  |  |  |  |  |  |  |  |  |  |  |  |  |  |  |  | 0.62 |  |
| Females |  |  |  |  | 16 | 80 |  |  |  |  | 13 | 68 |  |  |  |  | 10 | 67 |  |  |
| Males |  |  |  |  | 4 | 20 |  |  |  |  | 6 | 32 |  |  |  |  | 5 | 33 |  |  |
| **BMI (kg/m^2^)** | 31.31 | 4.46 | 30.35 | (27.87, 34.93) |  |  | 32.78 | 3.93 | 30.96 | (30.14, 35.66) |  |  | 32.53 | 3.21 | 32.47 | (30.17, 33.70) |  |  | 0.26 |  |
| **EQ-5D-5L utility** | 0.88 | 0.11 | 0.84 | (0.80, 1) |  |  | 0.81 | 0.13 | 0.84 | (0.75, 0.88) |  |  | 0.83 | 0.17 | 0.80 | (0.75, 1) |  |  | 0.33 |  |
| **EQ-5D VAS** | 75.65 | 16.68 | 75 | (62.5, 90) |  |  | 67.21 | 14.01 | 70 | (55, 75) |  |  | 66 | 17.85 | 60 | (55, 75) |  |  | 0.12 |  |
| BMI, body mass index; EQ-5D, EuroQol five-dimension questionnaire; IQR, interquartile range; kg, kilogram; m, meter; n, number; PP, Personalised Plan; PP+B, Personalised plan + Behavioural change; sd, standard deviation; VAS, Visual Analogue Scale.  ^Sample size was calculated with the aim to detect a 2.40 cm difference in waist circumference between groups with 80% power with a two-tailed α of 0.05, to allow for a 20% drop out. The targeted population size was not reached due to the impact of the COVID-19 pandemic. | | | | | | | | | | | | | | | | | | | |  |
|  | | | | | | | | | | | | | | | | | | | |  |

## Supplement 4: Outcomes of the PREVENTOMICS trials in Poland and the UK

**Table S3**: Trial outcomes (after 4 months) Poland

| **Variables** | **Effect in PP+B, means (SE)** | **Effect in PP, means (SE)** | **Effect in Control, means (SE)** | **Mean difference PP+B-Control (95% CI)** | **P-value** | **Mean difference PP-Control (95% CI)** | **P-value** |
| --- | --- | --- | --- | --- | --- | --- | --- |
|  |  |  |  |  |  |  |  |
| **BMI (kg/m^2^)^a^** | -1.03 (0.23)** | -0.63 (0.22)** | -0.82 (0.24)** | -0.20 (-0.86, 0.45) | 0.54 | 0.20 (-0.45, 0.85) | 0.55 |
| **EQ-5D utilities^b^** | -0.00 (0.01) | 0.01 (0.01) | 0.01 (0.01) | -0.01 (-0.04, 0.02) | 0.38 | 0.00 (-0.03, 0.03) | 0.91 |
| **EQ-5D VAS^b^** | 2.28 (1.70) | 4.54 (2.12)* | 4.42 (2.41) | -2.14 (-7.92, 3.65) | 0.47 | 0.12 (-6.16, 6.40) | 0.97 |
| ^a^ Linear mixed models were used: participants’ identification was included as random intercept and time of measurement (visit), intervention group, interaction between time and intervention, sex and age were included as fixed effects.  ^b^ Generalized estimation equations were used: sex, age, baseline HRQoL, time of measurement (visit) and intervention group (PP vs control), as well as the interaction between time and intervention, were included as fixed covariates.  BMI, body mass index; CI, confidence interval; EQ-5D, EuroQOL five-dimension questionnaire; kg, kilogram; m, meter; Personalised Plan; PP+B, Personalised plan + Behavioural change; SE, standard error; VAS, Visual Analogue Scale**.**  *p<0.05 significant change from baseline   **p<0.01 significant change from baseline | | | | | | | |

**Table S4**: Trial outcomes (after 4 months) UK

| **Variables** | **Effect in PP+B, means (SE)** | **Effect in PP, means (SE)** | **Effect in Control, means (SE)** | **Mean difference PP+B-Control (95% CI)** | **P-value** | **Mean difference PP-Control (95% CI)** | **P-value** |
| --- | --- | --- | --- | --- | --- | --- | --- |
|  |  |  |  |  |  |  |  |
| **BMI (kg/m^2^)^a^** | -1.31 (0.27)** | -0.84 (0.27)** | -0.51 (0.31) | -0.80 (-1.60, 0.00) | 0.05 | -0.33 (-1.14, 0.48) | 0.43 |
| **EQ-5D utilities^b^** | 0.01 (0.03) | 0.06 (0.02)** | -0.02 (0.04) | 0.03 (-0.07, 0.13) | 0.58 | 0.08 (-0.02, 0.17) | 0.11 |
| **EQ-5D VAS^b^** | 7.1 (3.78) | 8.11 (2.58)** | 9.67 (3.99)* | -2.57 (-13.34, 8.21) | 0.64 | -1.56 (-10.88, 7.76) | 0.74 |
| ^a^ Linear mixed models were used: participants’ identification was included as random intercept and time of measurement (visit), intervention group, interaction between time and intervention, sex and age were included as fixed effects.  ^b^ Generalized estimation equations were used: sex, age, baseline HRQoL, time of measurement (visit) and intervention group (PP vs control), as well as the interaction between time and intervention, were included as fixed covariates.  BMI, body mass index; CI, confidence interval; EQ-5D, EuroQOL five-dimension questionnaire; kg, kilogram; m, meter; Personalised Plan; PP+B, Personalised plan + Behavioural change; SE, standard error; VAS, Visual Analogue Scale**.**  *p<0.05 significant change from baseline   **p<0.01 significant change from baseline | | | | | | | |

## Supplement 5: Details on cost-effectiveness results

|  | Discounted | | | | | | Undiscounted | | | | | |
| --- | --- | --- | --- | --- | --- | --- | --- | --- | --- | --- | --- | --- |
|  | **PP** | | **Control** | | **Difference** | | **PP** | | **Control** | | **Difference** | |
| Effects | | | | | | | | | | | | |
| Life years | 19.36 |  | 19.372 |  | -0.013 |  | 35.613 |  | 35.654 |  | -0.041 |  |
| Life years with diabetes | 2.952 |  | 2.908 |  | 0.044 |  | 6.543 |  | 6.46 |  | 0.083 |  |
| Cum. Incident cases^a^ diabetes/1000 | 313.619 |  | 310.72 |  | 2.899 |  | 313.619 |  | 310.72 |  | 2.899 |  |
| Cum. Incident cases^a^ IHD/1000 | 328.097 |  | 327.171 |  | 0.926 |  | 328.097 |  | 327.171 |  | 0.926 |  |
| Cum. Incident cases^a^  stroke/1000 | 412.369 |  | 410.721 |  | 1.648 |  | 412.369 |  | 410.721 |  | 1.648 |  |
| QALYs | 16.510 |  | 16.525 |  | -0.015 |  | 29.365 |  | 29.405 |  | -0.040 |  |
| Costs (2020 £, PLN) | | | | | | | | | | | | |
| Diabetes | 2,037 | 10,183 | 2,005 | 10,023 | 32 | 161 | 6,147 | 30,735 | 6,070 | 30,352 | 77 | 383 |
| IHD | 3,409 | 17,044 | 3,388 | 16,939 | 21 | 104 | 12,855 | 64,276 | 12,799 | 63,997 | 56 | 279 |
| Stroke | 3,605 | 18,024 | 3,579 | 17,894 | 26 | 130 | 13,268 | 66,338 | 13,193 | 65,965 | 75 | 373 |
| Unrelated | 9,550 | 47,749 | 9,550 | 47,752 | -1 | -3 | 24,566 | 122,829 | 24,581 | 122,903 | -15 | -75 |
| Non-medical | 56,310 | 281,550 | 56,342 | 281,711 | -32 | -161 | 13,2661 | 663,305 | 132,827 | 664,137 | -166 | -832 |
| Intervention^a^ | 529 | 2,643 | 274 | 1,369 | 255 | 1,274 | 528 | 2,643 | 274 | 1,369 | 255 | 1,274 |
| Informal care | 1,152 | 5,761 | 1,153 | 5,766 | -1 | -4 | 7,372 | 36,858 | 7,393 | 36,965 | -21 | -107 |
| Productivity | 3,290 | 16,448 | 3,288 | 16,438 | 2 | 10 | 5,295 | 26,473 | 5,292 | 26,461 | 2 | 11 |
| TOTAL | 79,880 | 399,401 | 79,578 | 397,892 | 302 | 1,509 | 202,691 | 1,013,456 | 202,430 | 1,012,149 | 261 | 1,306 |
| *ICUR* |  |  |  |  | ***Control dominates*** | |  |  |  |  | **Control dominates** | |
| Cum., Cumulative; ICUR, incremental cost-utility ratio; IHD, Ischemic heart disease; PLN, Zloty; PP, Personalised Plan; PP+B, Personalised plan + Behavioural change; QALYs, quality-adjusted life years.  ^a^Not discounted | | | | | | | | | | | | |

**Table S5:** Details on cost-effectiveness results PP base-case Poland

**Table S6:** Details on cost-effectiveness results PP+B base-case Poland

|  | Discounted | | | | | | | Undiscounted | | | | | |
| --- | --- | --- | --- | --- | --- | --- | --- | --- | --- | --- | --- | --- | --- |
|  | **PP+B** | | **Control** | | **Difference** | | | **PP+B** | | **Control** | | **Difference** | |
| Effects | | | | | | | | | | | | | |
| Life years | 19.385 |  | 19.372 |  | 0.013 |  | | 35.694 |  | 35.654 |  | 0.04 |  |
| Life years with diabetes | 2.865 |  | 2.908 |  | -0.043 |  | | 6.379 |  | 6.46 |  | -0.081 |  |
| Cum. Incident cases^a^ diabetes/1000 | 307.865 |  | 310.72 |  | -2.855 |  | | 307.865 |  | 310.72 |  | -2.855 |  |
| Cum. Incident cases^a^ IHD/1000 | 326.253 |  | 327.171 |  | -0.919 |  | | 326.253 |  | 327.171 |  | -0.919 |  |
| Cum. Incident cases^a^  stroke/1000 | 409.089 |  | 410.721 |  | -1.632 |  | | 409.089 |  | 410.721 |  | -1.632 |  |
| QALYs | 16.536 |  | 16.525 |  | 0.011 |  | | 29.441 |  | 29.405 |  | 0.036 |  |
| Costs (2020 £, PLN) | | | | | | | | | | | | | |
| Diabetes | 1,973 | 9,866 | 2,005 | 10,023 | -31 | -157 | | 5,995 | 29,976 | 6,070 | 30,352 | -75 | -376 |
| IHD | 3,367 | 16,836 | 3,388 | 16,939 | -21 | -103 | | 12,744 | 63,720 | 12,799 | 63,997 | -55 | -276 |
| Stroke | 3,553 | 17,766 | 3,579 | 17,894 | -26 | -128 | | 13,119 | 65,597 | 13,193 | 65,965 | -74 | -368 |
| Unrelated | 9,551 | 47,756 | 9,550 | 47,752 | 1 | 3 | | 24,595 | 122,977 | 24,581 | 122,903 | 15 | 74 |
| Non-medical | 56,374 | 281,870 | 56,342 | 281,711 | 32 | 159 | | 132,992 | 664,958 | 132,827 | 664,137 | 164 | 821 |
| Intervention^a^ | 547 | 2,733 | 274 | 1,369 | 273 | 1,364 | | 547 | 2,733 | 274 | 1,369 | 273 | 1,364 |
| Informal care | 1,154 | 5,770 | 1,153 | 5,766 | 1 | 4 | | 7,414 | 37,072 | 7,393 | 36,965 | 21 | 106 |
| Productivity | 3,286 | 16,428 | 3,288 | 16,438 | -2 | -10 | | 5,290 | 26,450 | 5,292 | 26,461 | -2 | -11 |
| TOTAL | 79,805 | 399,025 | 79,578 | 397,892 | 227 | 1,133 | | 202,697 | 1,013,483 | 202,430 | 1,012,149 | 267 | 1,334 |
| *ICUR* |  |  |  |  | **20,404** | | **102,018** |  |  |  |  | **7,411** | **37,056** |
| Cum., Cumulative; ICUR, incremental cost-utility ratio; IHD, Ischemic heart disease; PLN, Zloty; PP, Personalised Plan; PP+B, Personalised plan + Behavioural change; QALYs, quality-adjusted life years.  ^a^Not discounted | | | | | | | | | | | | | |

| **Table S7:** Details on cost-effectiveness results PP base-case UK | | | | | | | |
| --- | --- | --- | --- | --- | --- | --- | --- |
|  | **Discounted** | | | | **Undiscounted** | | |
|  | | **PP** | **Control** | **Difference** | **PP** | **Control** | **Difference** |
| **Effects** | | | | | | | |
| **Life years** | | 19.776 | 19.762 | 0.014 | 36.577 | 36.53 | 0.047 |
| **Life years with diabetes** | | 1.596 | 1.627 | -0.031 | 3.841 | 3.905 | -0.065 |
| **Cum. Incident cases^a^ diabetes/1000** | | 212.343 | 215.196 | -2.853 | 212.343 | 215.196 | -2.853 |
| **Cum. Incident cases^a^ IHD/1000** | | 343.553 | 344.921 | -1.368 | 343.553 | 344.921 | -1.368 |
| **Cum. Incident cases^a^  stroke/1000** | | 342.263 | 343.577 | -1.314 | 342.263 | 343.577 | -1.314 |
| **QALYs** | | 16.019 | 15.979 | 0.04 | 28.732 | 28.662 | 0.069 |
| **Costs (2020 £)** | | | | | | | |
| **Diabetes** | | 2,599 | 2,649 | -51 | 6,350 | 6,456 | -106 |
| **IHD** | | 7,471 | 7,527 | -56 | 20,025 | 20,144 | -119 |
| **Stroke** | | 4,706 | 4,744 | -38 | 13,493 | 13,576 | -83 |
| **Unrelated** | | 42,435 | 42,393 | 43 | 91,843 | 91,684 | 159 |
| **Non-medical** | | 222,353 | 222,209 | 145 | 401,992 | 401,533 | 459 |
| **Intervention** | | 1,157 | 718 | 439 | 1,157 | 718 | 439 |
| **Informal care** | | 9,181 | 9,169 | 12 | 31,045 | 30,961 | 84 |
| **Productivity** | | 9,448 | 9,453 | -5 | 13,151 | 13,155 | -4 |
| **TOTAL** | | 299,351 | 298,862 | 489 | 579,057 | 578,228 | 829 |
| ***ICUR*** | |  |  | **12,222** |  |  | **12,014** |
| Cum.**,** Cumulative**;** ICUR**,** incremental cost-utility ratio**;** IHD**,** Ischemic heart disease**;** PP**,** Personalised Plan**;** PP+B, Personalised plan + Behavioural change**;** QALYs**,** quality-adjusted life years. | | | | | | | |
| ^a^Not discounted | | | | | | | |

| **Table S8:** Details on cost-effectiveness results PP+B base-case UK | | | | | | | | |
| --- | --- | --- | --- | --- | --- | --- | --- | --- |
|  | | **Discounted** | | | **Undiscounted** | | | |
|  | **PP+B** | | **Control** | **Difference** | | **PP+B** | **Control** | **Difference** |
| **Effects** | | | | | | | | |
| **Life years** | 19.796 | | 19.762 | 0.034 | | 36.642 | 36.53 | 0.112 |
| **Life years with diabetes** | 1.552 | | 1.627 | -0.075 | | 3.751 | 3.905 | -0.155 |
| **Cum. Incident cases^a^ diabetes/1000** | 208.368 | | 215.196 | -6.828 | | 208.368 | 215.196 | -6.828 |
| **Cum. Incident cases^a^ IHD/1000** | 341.617 | | 344.921 | -3.304 | | 341.617 | 344.921 | -3.304 |
| **Cum. Incident cases^a^  stroke/1000** | 340.409 | | 343.577 | -3.168 | | 340.409 | 343.577 | -3.168 |
| **QALYs** | 16.023 | | 15.979 | 0.044 | | 28.774 | 28.662 | 0.112 |
| **Costs (2020 £)** | | | | | | | | |
| **Diabetes** | 2,529 | | 2,649 | -121 | | 6,203 | 6,456 | -254 |
| **IHD** | 7,393 | | 7,527 | -134 | | 19,858 | 20,144 | -286 |
| **Stroke** | 4,654 | | 4,744 | -90 | | 13,377 | 13,576 | -198 |
| **Unrelated** | 42,495 | | 42,393 | 102 | | 92,065 | 91,684 | 382 |
| **Non-medical** | 222,554 | | 222,209 | 345 | | 402,631 | 401,533 | 1,098 |
| **Intervention** | 1,175 | | 718 | 457 | | 1,175 | 718 | 457 |
| **Informal care** | 9,198 | | 9,169 | 29 | | 31,162 | 30,961 | 201 |
| **Productivity** | 9,441 | | 9,453 | -12 | | 13,145 | 13,155 | -11 |
| **TOTAL** | 299,438 | | 298,862 | 576 | | 579,617 | 578,228 | 1,389 |
| ***ICUR*** |  | |  | **13,006** | |  |  | **12,402** |
| Cum.**,** Cumulative**;** ICUR**,** incremental cost-utility ratio**;** IHD**,** Ischemic heart disease**;** PP**,** Personalised Plan**;** PP+B, Personalised plan + Behavioural change**;** QALYs**,** quality-adjusted life years. | | | | | | | | |
| ^a^Not discounted | | | | | | | | |

## Supplement 6: CE-plane comparing with ‘no intervention’


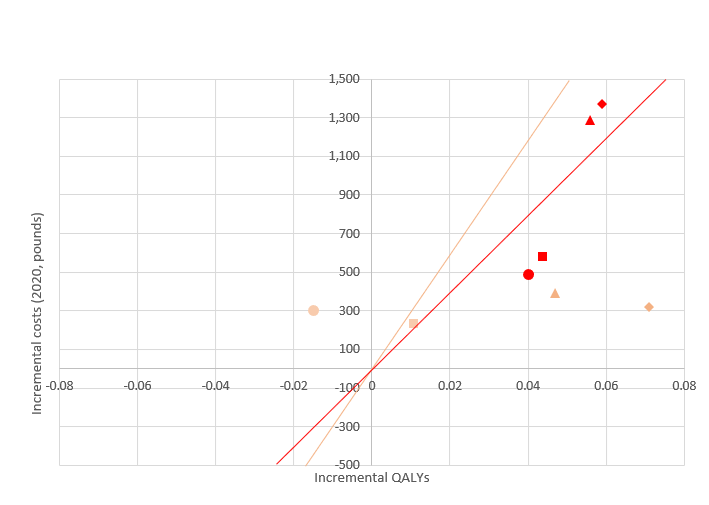


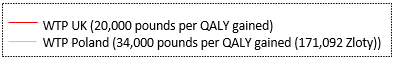

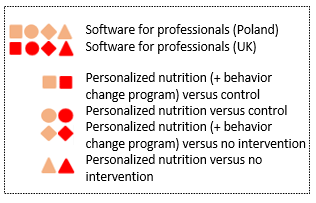


## Supplement 7: Model inputs for scenario analyses: effectiveness results obtained from the PREVENTOMICS trials in Poland and the UK^[[1]](#footnote-1)^

**Table S9:** model inputs scenario analyses

| **Parameter** | **Deterministic value** | | **Sensitivity analysis range (CI or assumption)** | | **Deterministic value** | | **Sensitivity analysis range (CI or assumption)** | |
| --- | --- | --- | --- | --- | --- | --- | --- | --- |
|  | **Poland** | | | | **UK** | | | |
|  | **Female** | **Male** | **Female** | **Male** | **Female** | **Male** | **Female** | **Male** |
| **General** | | | | | | | | |
| **Baseline characteristics:** |  |  |  |  |  |  |  |  |
| BMI, at start | 31.98 | 33.07 |  |  | 31.73 | 33.31 |  |  |
| Age, at start | 44.4 | 41.51 |  |  | 44.52 | 47.09 |  |  |
| **Effects^a^** | | | | | | | | |
| **PP+B vs. Control:** Effect BMI, kg/m2 (SE) | 0.42 (0.29) | -1.55 (0.52)** | -0.14, 0.99 | -2.56, -0.53 | -0.76 (0.50) | -0.65 (0.67) | -1.75, 0.22 | -1.97, 0.66 |
| **PP vs. Control:**  Effect BMI, kg/m2 (SE) | 0.80 (0.28)** | -1.54 (0.56)** | 0.25, 1.35 | -2.64, -0.44 | 0.05 (0.53) | -1.13 (0.61) | -0.98, 1.08 | -2.32, 0.06 |
| **PP+B vs. Control:** Effect Qol, EQ-5D utilities (SE) | -0.01 (0.02) | -0.02 (0.02) | -0.05, 0.03 | -0.06, 0.01 | 0.02 (0.06) | 0.08 (0.09) | -0.11, 0.14 | -0.10, 0.26 |
| **PP vs. Control:**  Effect Qol, EQ-5D utilities (SE) | 0.01 (0.02) | -0.01 (0.03) | -0.03, 0.04 | -0.06, 0.04 | 0.07 (0.06) | 0.10 (0.07) | -0.05, 0.19 | -0.05, 0.24 |
| ^a^Corrected for baseline BMI/utility  **p<0.01 significant change from baseline  BMI, body mass index; CI, confidence interval; EQ-5D, EuroQOL five-dimension questionnaire; kg, kilograms; m, meter; PP**,** Personalised Plan**;** PP+B, Personalised plan + Behavioural change; QoL, quality of life; SE, standard error. | | | | | | | | |

## Supplement 8: Tornado diagrams incremental QALYs and Costs

AA


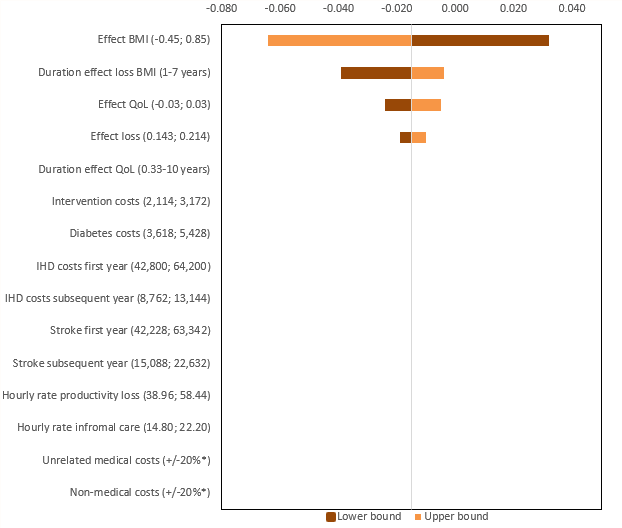

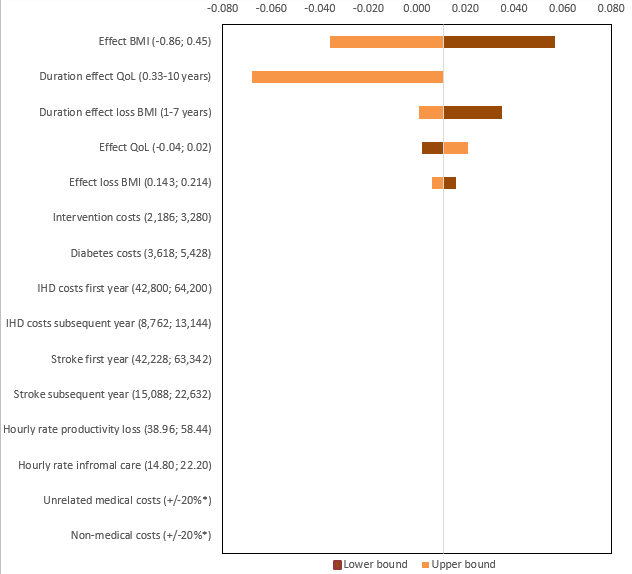


B


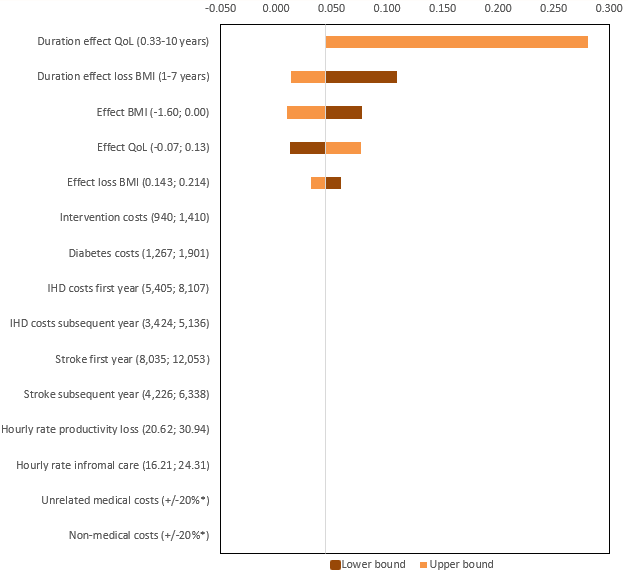


D

C


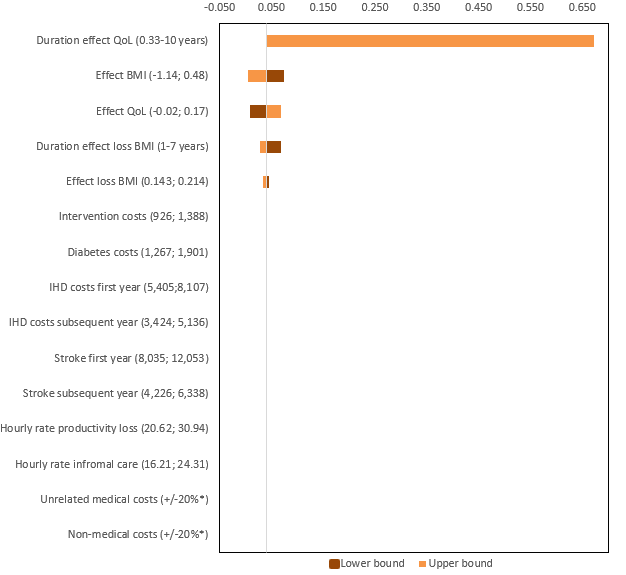


**Figure S1:** Tornado diagrams for change in incremental QALYs using lower and upper bounds of parameters (A) for Poland in the PP+B group, (B) for Poland in the PP group, (C) for the UK in the PP+B group, and (D) for the UK in the PP group. BMI, Body Mass Index; IHD, ischemic heart disease; QoL, quality of life. *No fixed number, since costs differ by sex and age.


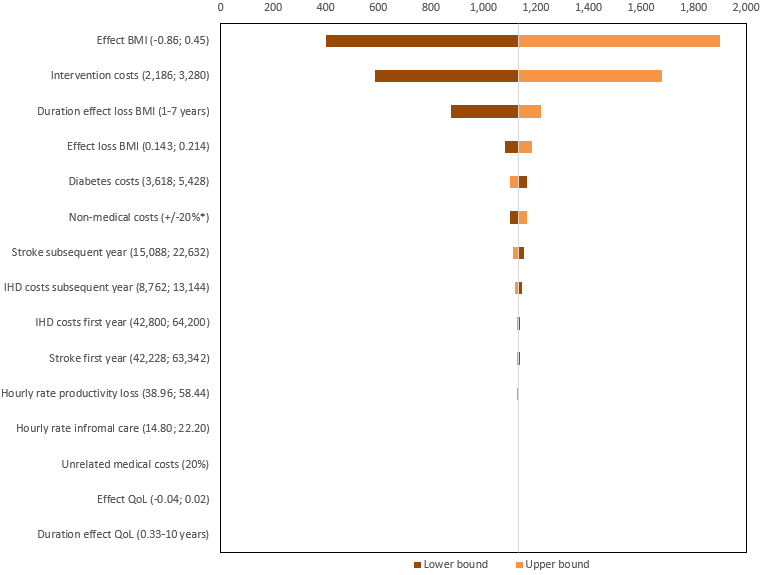

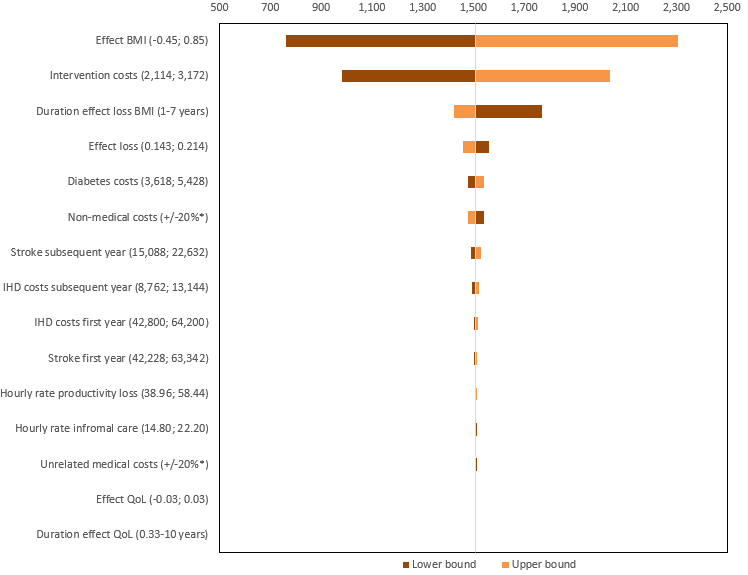


AA

B

D

C


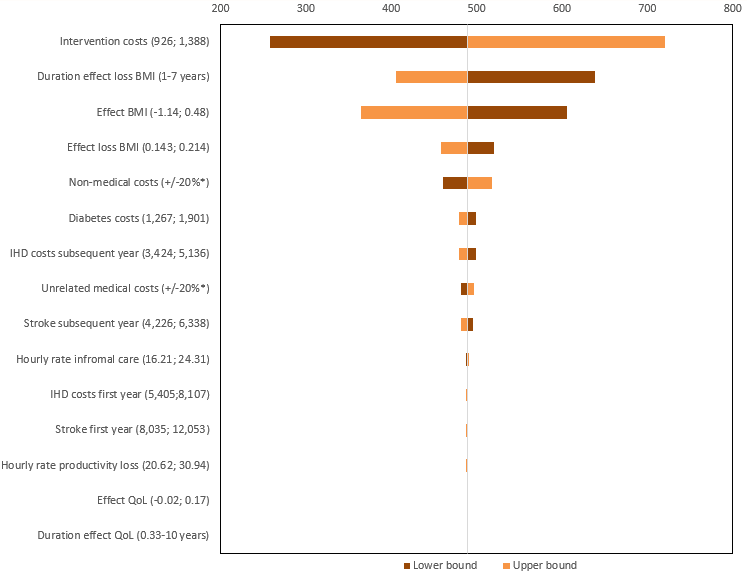

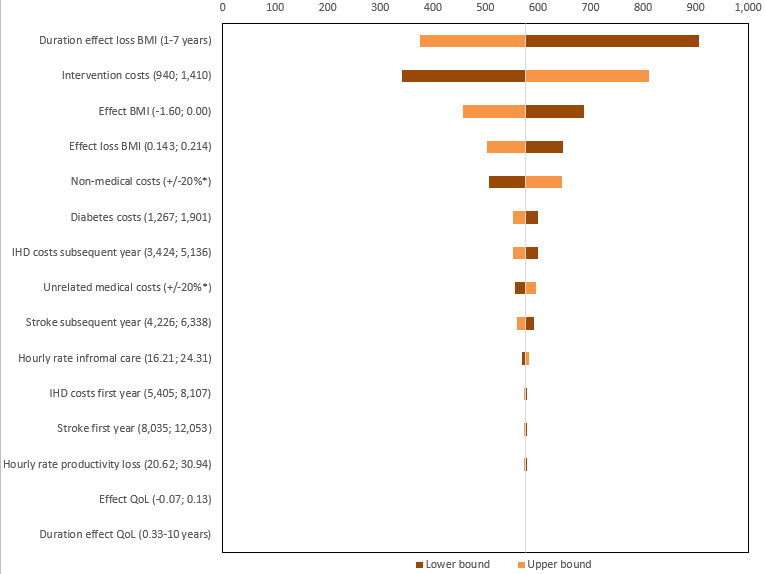


**Figure S2:** Tornado diagram for change in incremental costs using lower and upper bounds of parameters (A) for Poland in the PP+B group (in PLN), (B) for Poland in the PP group (in PLN), (C) for the UK in the PP+B group (in £), and (D) for the UK in the PP group (in £).

## Supplement 9: CEAC’s

AA

B


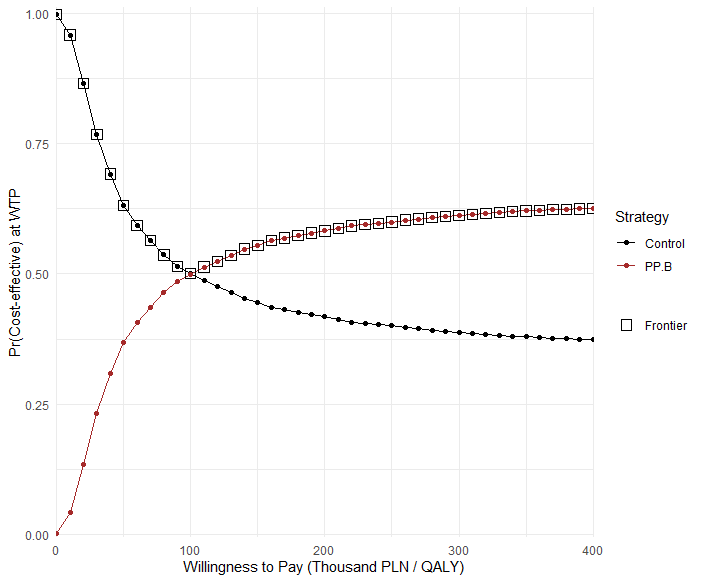


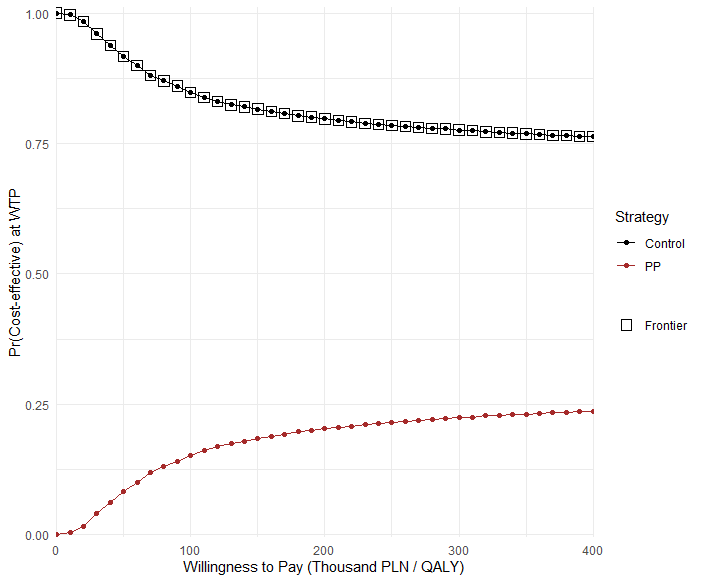


C

D


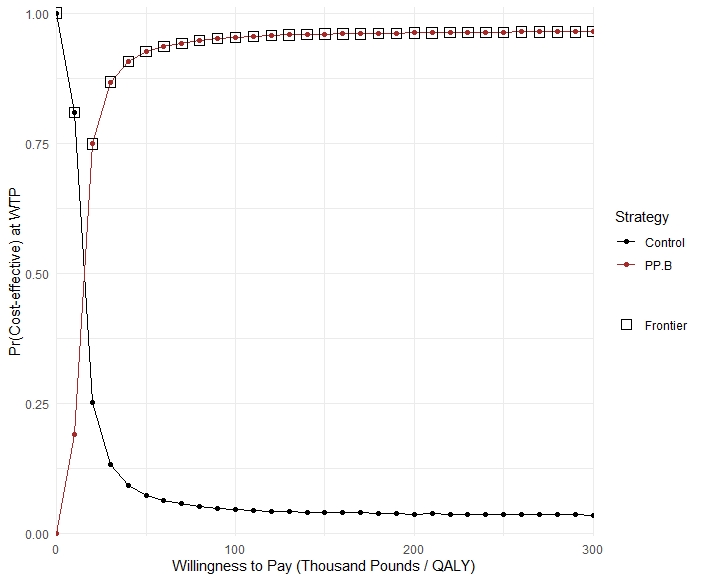

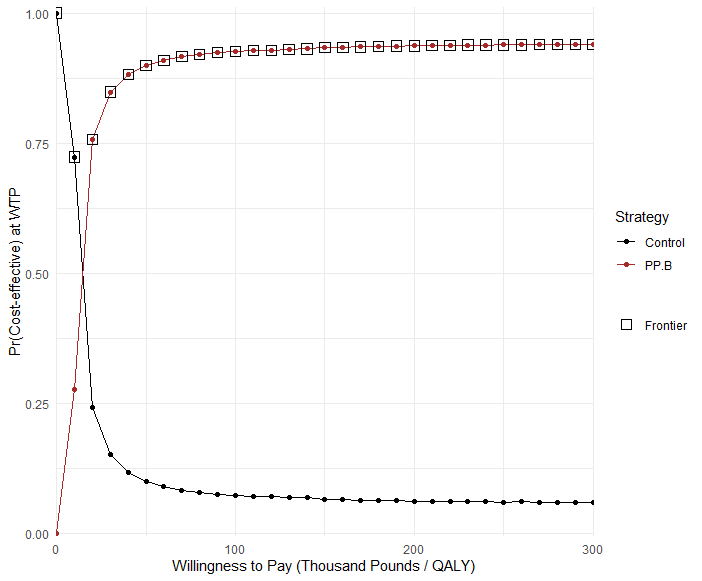


**Figure S3:** Cost-effectiveness acceptability curve plot for (A) Poland: PP+B versus control, (B) Poland: PP versus control, (C) UK: PP+B versus control, and (D) UK: PP versus control. PLN, Zloty; PP, Personalised Plan; PP+B, Personalised plan + Behavioural change; QALY, quality-adjusted life year; WTP, willingness-to-pay.

## References

1. Boshuizen HC, Lhachimi SK, van Baal PHM, et al. The DYNAMO-HIA Model: An Efficient Implementation of a Risk Factor/Chronic Disease Markov Model for Use in Health Impact Assessment (HIA). *Demography*. 2012;49(4):1259-1283. doi:10.1007/s13524-012-0122-z

2. DYNAMO-HIA project. Workpackage 7: Overweight and Obesity. Report on data collection for overweight and obesity prevalence and related relative risks. Accessed March 12, 2020. https://www.dynamo-hia.eu/sites/default/files/2018-04/BMI_WP7-datareport_20100317.pdf

3. Eurostat. Health care expenditure by financing scheme: HLTH_SHA11_HF. Published 2022. Accessed March 10, 2022. https://ec.europa.eu/eurostat/databrowser/view/HLTH_SHA11_HF__custom_3037914/default/table?lang=en

4. Kalseth J, Anthun KS, Forma L. Health care and long-term care costs by age and proximity to death in a publicly funded universal system: A descriptive study of population data. *Nordic Journal of Health Economics*. 2020;8(1):31-45. doi:10.5617/njhe.7070

5. OECD (2022). Inflation (CPI) (indicator). Accessed July 19, 2022. doi: 10.1787/eee82e6e-en

6. Hoogendoorn M, Galekop MMJ, van Baal P. The lifetime health and economic burden of obesity in five European countries: what is the potential impact of prevention? *Diabetes Obes Metab*. 2023;28(8). doi:10.1111/dom.15116

7. Aune D, Sen A, Prasad M, et al. BMI and all cause mortality: Systematic review and non-linear dose-response meta-analysis of 230 cohort studies with 3.74 million deaths among 30.3 million participants. *BMJ (Online)*. 2016;353. doi:10.1136/bmj.i2156

8. GBD 2015 Obesity Collaborators. Health Effects of Overweight and Obesity in 195 Countries over 25 years. *N Engl J Med*. 2017;377(1):13-27.

1. Only the results of trials’ observed effectiveness are shown, since all other model inputs (e.g., general/costs) were equal to the base-case scenario. [↑](#footnote-ref-1)
